# The Endoplasmic Reticulum-Resident Chaperone Heat Shock Protein 47 Protects the Golgi Apparatus from the Effects of *O*-Glycosylation Inhibition

**DOI:** 10.1371/journal.pone.0069732

**Published:** 2013-07-29

**Authors:** Shingo Miyata, Tatsunori Mizuno, Yoshihisa Koyama, Taiichi Katayama, Masaya Tohyama

**Affiliations:** 1 Division of Molecular Brain Science, Research Institute of Traditional Asian Medicine, Kinki University, Osakasayama, Osaka, Japan; 2 Department of Anatomy and Neuroscience, Graduate School of Medicine, Osaka University, Suita, Osaka, Japan; 3 Department of Child Development and Molecular Brain Science, United Graduate School of Child Development, Osaka University, Kanazawa University and Hamamatsu University School of Medicine, Suita, Osaka, Japan; The University of Texas MD Anderson Cancer Center, United States of America

## Abstract

The Golgi apparatus is important for the transport of secretory cargo. Glycosylation is a major post-translational event. Recognition of *O*-glycans on proteins is necessary for glycoprotein trafficking. In this study, specific inhibition of *O*-glycosylation (Golgi stress) induced the expression of endoplasmic reticulum (ER)-resident heat shock protein (HSP) 47 in NIH3T3 cells, although cell death was not induced by Golgi stress alone. When HSP47 expression was downregulated by siRNA, inhibition of *O*-glycosylation caused cell death. Three days after the induction of Golgi stress, the Golgi apparatus was disassembled, many vacuoles appeared near the Golgi apparatus and extended into the cytoplasm, the nuclei had split, and cell death assay-positive cells appeared. Six hours after the induction of Golgi stress, HSP47-knockdown cells exhibited increased cleavage of Golgi-resident caspase-2. Furthermore, activation of mitochondrial caspase-9 and ER-resident unfolded protein response (UPR)-related molecules and efflux of cytochrome c from the mitochondria to the cytoplasm was observed in HSP47-knockdown cells 24 h after the induction of Golgi stress. These findings indicate that (i) the ER-resident chaperon HSP47 protected cells from Golgi stress, and (ii) Golgi stress-induced cell death caused by the inhibition of HSP47 expression resulted from caspase-2 activation in the Golgi apparatus, extending to the ER and mitochondria.

## Introduction

The cellular locations of major cytoplasmic organelles, such as the nucleus, endoplasmic reticulum (ER), Golgi apparatus, and mitochondria, are unique, and the organelles have specific functions based on their unique location. It is well established that dysfunction of the nucleus, ER, and mitochondria induce cell death and pro-apoptotic signals [1–3]. Several studies have analyzed stimuli (stress) that induce cell death and the expression of apoptosis-related molecules via mitochondrial and nuclear dysfunction [4–7]. Recently, ER dysfunction-induced cell death was reported, particularly in neurodegeneration [8–18]. This organelle exhibits specific mechanisms for counteracting stress stimuli [19–23].

The Golgi apparatus is an essential organelle for processing and sorting lipids and proteins [24–26], and it is also important for the transport of secretory cargo. Glycosylation is a major post-translational event, and the recognition of *O*-glycans on proteins is required for glycoprotein trafficking [24,25]. Such an event occurs through sorting in the trans-Golgi network, and glycoprotein trafficking is disturbed when *O*-glycosylation is inhibited [27–30]. Thus, inhibition of *O*-glycosylation is an effective stimulus for the Golgi apparatus (so called Golgi stress), although several non-specific stimuli, such as osmotic stress, change in temperature, change in pH, and change in ion content, also stress the Golgi apparatus [31–34]. However, in contrast to the ER and mitochondria, specific inhibition of *O*-glycosylation alone (specific Golgi stress) has not been fully investigated in relation to cell death. Only a few specific *O*-glycosylation inhibitors have been reported [27,29,30,35]. In the present study, benzyl 2-acetamido-2-deoxy-α-d-galactopyranoside (GalNAc-bn) was used as an *O*-glycosylation inhibitor [30,35,36,37]. We show that in NIH3T3 cells Golgi stress induced by GalNAc-bn influences ER function and induced cell death via inhibition of the ER-resident chaperone heat shock protein 47 (HSP47).

## Materials and Methods

### Chemicals and antibodies

The following antibodies were used: anti-HSP47 monoclonal antibody (mAb), anti-Grp78 mAb (10C3), anti-Grp78 polyclonal antibody (pAb) (Assay Designs, Inc., Ann Arbor, MI, USA); anti-GAPDH mAb, anti-GADD153 (CHOP) pAb, anti-cytochrome *c* pAb (Santa Cruz Biotechnology, Santa Cruz, CA, USA); anti-GFP pAb (MBL International Co., Nagoya, Japan); anti-caspase-2 pAb (R&D Systems, Inc., Minneapolis, MN, USA); anti-type I collagen pAb; anti-type IV collagen pAb (Millipore, MA, USA); anti-GM130 mAb (BD Transduction Laboratories, Franklin Lakes, NJ, USA); anti-calnexin pAb, anti-IRE1α mAb, anti-phospho-PKR-like ER kinase (PERK) mAb, anti-caspase-9 mAb (C9), anti-BclxL pAb (Cell Signaling Technology, Beverly, MA, USA); anti-caspase-2 pAb, anti-HADHA pAb and anti-ATF6 mAb (Abcam Inc., Cambridge, MA, USA). The chemical reagents used in this study included benzyl 2-acetamido-2-deoxy-α-d-galactopyranoside (GalNAc-bn), thapsigargin (Tg), tunicamycin(TM), staurosporine (STS), etoposide (Eto), and monensin (Sigma Chemical Co., St. Louis, MO, USA).

### Cell culture

The mouse embryonic fibroblast cell line NIH3T3 (RBRC-RCB2767; Riken BRC Cell bank, Tsukuba, Japan) was maintained in tissue culture dishes (Nunc, Roskilde, Denmark) in Dulbecco’s modified Eagle’s medium (DMEM; Life Technologies Inc., Carlsbad, CA, USA) with 10% heat-inactivated fetal bovine serum (FBS) at 37°C in an atmosphere of 95% air/5% CO_2_. The human colorectal cancer cell line Colo 205 (ATCC CCL-222; American Type Culture Collection, Rockville, MD, USA) was maintained in RPMI-1640 medium with 10% FBS. We used Tm (1 µg/mL) and Tg (1 µM) as ER stress inducers for the indicated durations. These cells were transfected using Lipofectamine 2000 reagent or Lipofectamine RNAiMAX reagent (Life Technologies Inc.), according to the manufacturer’s instructions.

### Reverse transcriptase (RT) reaction and real time PCR

Total RNA was prepared using ISOGEN (NipponGene, Toyama, Japan), according to the manufacturer’s instructions. The extracted total RNA was reverse transcribed using oligo(dT) 12–18 primers and SuperScript III RNase H-Reverse Transcriptase (Life Technologies Inc.). Real-time PCR was performed on an ABI PRISM 7900HT Sequence Detection System using the SYBR Green PCR Master Mix (Life Technologies Inc.). The resulting cDNA (50 ng) was then mixed with 0.1 µM primers and 10 µL of the master mix in a 20-µL final volume. To quantify *Hsp47* gene expression levels, the following primers were used: forward, 5′-ATCAATCAGCAATGCCTCCTGC-3′, and reverse, 5′-ATGGCATGGACTGTGGTCATG-3′. The primers for GAPDH were as follows: forward, 5′-ATCAATCAGCAATGCCTCCTGC-3′; reverse, 5′-ATGGCATGGACTGTGGTCATG-3′.

### Lectin blotting and immunoblotting

Cells were lysed with a buffer containing 20 mM Tris-HCl (pH 7.8), 0.15 M NaCl, 1 mM EDTA, 0.2% NP-40, and 1 mM PMSF. Cell debris was clarified by centrifugation for 10 min at 16,000 × *g* at 4°C. Cell lysates were normalized for protein content using the Dc Protein Assay (BioRad Laboratories, Hercules, CA, USA). The normalized proteins were incubated in SDS-loading buffer for 30 min at 4°C, separated by SDS-PAGE, and transferred to a polyvinylidene difluoride (PVDF) membrane (Millipore). Membranes were incubated with Blocking Reagent (GE Healthcare, Pollards Wood, UK) for 1 h at room temperature and incubated with lectin peanut agglutinin (PNA)-biotin (Honen Corporation, Tokyo, Japan) or primary antibodies in blocking buffer overnight at 4°C in phosphate-buffered saline (PBS) containing 0.3% Tween 20 (PBS-T). Immunodetection was performed using the ECL Western Blotting Detection System (GE Healthcare) with streptavidin-HRP (DAKO, Glostrup, Denmark) or peroxidase-coupled secondary antibodies, according to the manufacturer’s instructions.

### Knockdown experiment using siRNA

Stealth siRNA against mouse *Hsp47* (5′-**GGGUCUUGUGUCACUGGGUGGUAAA**-3′) and negative control duplexes (scrambled siRNA for mouse *Hsp47*, 5′-**GGGUGUUACUGGGUCGGUGUCUAAA**-3′) were provided by Life Technologies Inc. Commercially available siRNAs for caspase-2 from Santa Cruz Biotechnology, Inc. were also used in this study according to the manufacturer’s instructions. Briefly, NIH3T3 cells and Colo 205 cells were transfected with 10 µl Caspase-2 siRNA, 200 pM Hsp47 siRNA or a scrambled siRNA using Lipofectamine RNAiMAX (Life Technologies Inc.) for 2 d, according to the manufacturer’s instructions. *Hsp47* and *caspase-2* knockdown were confirmed by RT-PCR and/or western blotting.

### Plasmid construction

A green fluorescent protein (GFP)-HA-fused human HSP47 (mouse *Hsp47* siRNA-insensitive human HSP47) plasmid was constructed using the pcDNA3.1 eukaryotic expression vector (Life Technologies Inc.). To construct the negative control, the GFP moiety was amplified from pEGFP-C1 (Takara Bio Inc., Kyoto, Japan) using the following primer pair: 5′-**GCTAGCGCCACCATGGTGAGCAAGGGCGAGGAGCTG**-3′ (forward) and 5′-**GATATCCTTGTACAGCTCGTCCATGCC**-3′ (reverse). Human HSP47 was amplified from a human brain cDNA library using PCR. HSP47 was amplified using rTaq DNA polymerase (Takara Bio Inc.) with the following primer set: 5′-**GATATCCGCTCCCTCCTGCTTCTCAGCGCC**-3′ (forward) and 5′-**GCGGCCGCCTATAACTCGACTCGCATCTTGTC**-3′ (reverse). The amplified fragments were TA-cloned into the pGEM-T vector (Promega Corp., Madison, WI, USA) and sequenced from the T7 or SP6 promoter. The *Nhe*I- and *Eco*RV-excised GFP and the *Eco*RV- and *Not*I-excised HSP47 were ligated into a pcDNA3.1 vector containing *Nhe*I and *Not*I sites (GFP-fused HSP47). pCAGGS-Bcl-xL was kindly gifted by Y. Eguchi and Y. Tsujimoto (Molecular Genetics, Osaka University Medical School, Japan). The pCAGGS and pEGFP plasmids were used as negative controls. The pcDNA3.1 plasmid was used as a neomycin-resistant gene expression vector.

### Subcellular fractionation

NIH3T3 cells in 0.25 M sucrose buffer were homogenized by 10 up-and-down strokes in a Teflon-glass motorized homogenizing vessel. Debris and nuclei were removed by centrifugation at 700 × *g* for 10 min at 4°C, and the supernatant (postnuclear homogenate) was centrifuged for 10 min at 5,000 × *g* at 4°C. The resulting pellet was resuspended in 10 µL of ice-cold lysis buffer (containing 0.1% NP-40, 20 mM Tris-HCl [pH 7.8], 0.15 M NaCl, 1 mM EDTA, and a protease inhibitor cocktail) to obtain the crude mitochondrial fraction. The supernatant was concentrated using a VIVASPIN 500 column (Sartorius Stedim Biotech, Göttingen, Germany) to obtain the crude cytoplasmic fraction. All steps were performed at 4°C. Cytochrome *c* efflux from the mitochondria to the cytoplasm was examined by western blot analysis of subcellular fractions. Contamination of mitochondria in the cytoplasmic fraction was determined by immunoblotting for HADHA, a protein specific to the mitochondria.

### Western blot analysis

Western blot analysis was performed as previously described [38]. Immunodetection was performed using the ECL Western Blotting Detection System (GE Healthcare) with peroxidase-coupled secondary antibodies according to the manufacturer’s instructions.

### Immunocytochemistry

NIH3T3 and Colo 205 cells were cultured in 4-well Lab-Tek Chamber Slides (Nunc) and treated with 2 mM GalNAc-bn or the same volume of DMSO for 10 d or with 10 mM GalNAc-bn for 1 d. These cells were fixed with 4% paraformaldehyde for 1 h at room temperature, then blocked for 1 h in 5% goat serum, 0.1% bovine serum albumin, and 0.1% Triton-X in PBS at room temperature, incubated with primary antibodies for over 12 h at 4°C, washed with PBS-T for 30 min, and treated with secondary antibodies. Fluorescence was analyzed on a Zeiss Axiovert 100 microscope (Carl Zeiss, Oberkochen, Germany).

### TUNEL assay

Cell death was assessed with the TMR Red In Situ Cell Death Detection Kit (Roche Ltd., Konzern-Hauptsitz, Schweiz), and cells were observed under a fluorescence microscope. Phenylindole dihydrochloride (DAPI) images (displaying all nuclei) were overlaid with TUNEL-stained images to enumerate the different cell populations (based on counting 100 nuclei). TUNEL-positive cells are expressed as a percentage of total DAPI-positive cells. For each experiment, at least 5 fields were examined by an individual who was blind to the experimental protocol.

### Electron microscopy

NIH3T3 cells were fixed at room temperature for 1 h in 0.1 M phosphate buffer (PB) containing 2.5% glutaraldehyde and 2% paraformaldehyde. These cells were rehydrated and rinsed in 0.1 M PB. Thereafter, the cells were post-fixed in 1% OsO_4_ at room temperature for 1 h, dehydrated in a graded ethanol series, and embedded in epon resin (Quetol 812; Nisshin EM Co, Tokyo, Japan). Areas containing several cells were block-mounted in epoxy resin using the direct epoxy-resin embedding method [16] and cut into 80-nm sections. The sections were counterstained with uranyl acetate and lead citrate and were analyzed using a Hitachi H-7650 transmission electron microscope.

## Results

### Evaluation of GalNAc-bn-induced *O*-glycosylation inhibition

**Figure 1:**
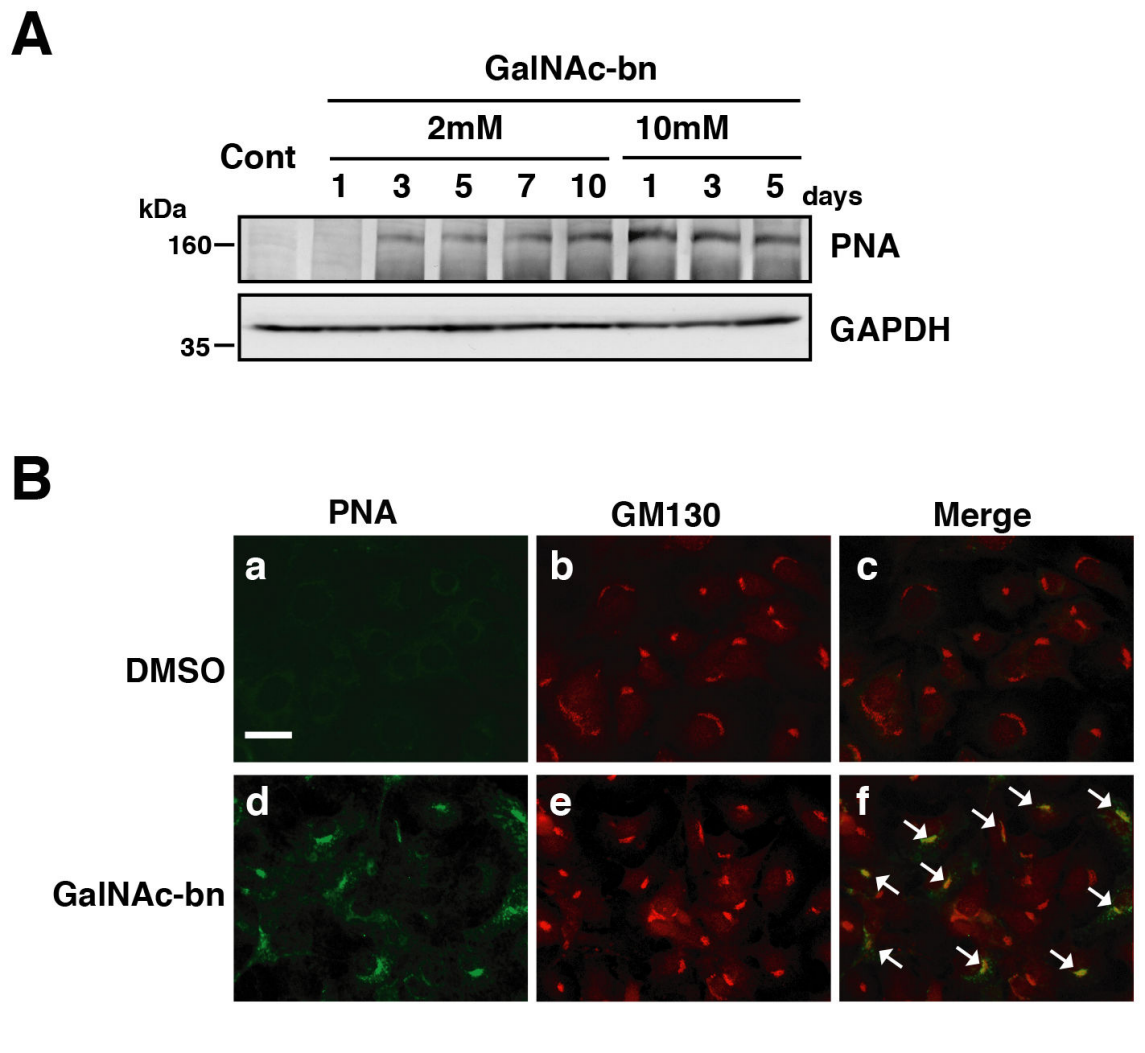
GalNAc-bn treatment decreased *O*-glycosylation in NIH3T3 cells. (A) Western blot analysis of PNA lectin binding (a marker for *O*-glycosylation levels) in NIH3T3 cells. Cells were treated with 2 mM or 10 mM GalNAc-bn for 10 d. (B) PNA immunoreactivity overlapped with the localization of the Golgi apparatus during GalNAc-bn treatment (arrow). GalNAc-bn-treated cells were observed at 24 h after stimulation. Scale bar: 30 µm.

In this study, GalNAc-bn was used as an *O*-glycosylation inhibitor [23,30,35,36]. First, we determined an appropriate GalNAc-bn concentration that would inhibit *O*-glycosylation in NIH3T3 cells by analyzing peanut agglutinin (PNA) binding to *O*-glycosylation-inhibited sites as a marker for the inhibition of *O*-glycosylation [27,37,39]. When the cells were exposed to 2 mM GalNAc-bn, a new weak PNA band appeared 3 d after treatment, which gradually increased in intensity up to day 10 post-treatment (Figure 1A). However, when NIH3T3 cells were exposed to 10 mM GalNAc-bn, a new strong PNA band was detected 1 d after treatment (Figure 1A). The effects of 10 mM GalNAc-bn on *O*-glycosylation inhibition were stable because the high-intensity PNA band was detected up to day 5 post-treatment (Figure 1A). Furthermore, *O*-glycosylation inhibition in response to a 1-d treatment with 10 mM GalNAc-bn was also observed in human colon adenocarcinoma Colo 205 cells, one of the most heavily *O* -glycosylated cell lines [40] (Figure S1A).

These findings indicate that 10 mM GalNAc-bn treatment resulted in stable and maximum inhibition of *O*-glycosylation 1 d after treatment, whereas the effects of 2 mM GalNAc-bn appeared gradually after 3 d of treatment. Although it was thought that various optimum concentrations were required for different cell lines and conditions, to obtain comprehensible results in the present study using NIH3T3 cells, we chose a 1-d treatment of NIH3T3 cells with 10 mM GalNAc-bn to inhibit *O*-glycosylation in subsequent experiments.

### Identification of the site of GalNAc-bn-induced inhibition of *O*-glycosylation

If the inhibition of *O*-glycosylation by GalNAc-bn occurred in the Golgi apparatus, the binding complex comprising PNA and *O*-glycosylation-inhibited sites would be detected in the Golgi apparatus. We sought to clarify this issue using NIH3T3 cells and Colo 205 cells.

To investigate whether PNA binding complexes were localized in the Golgi apparatus, we performed double immunostaining with antibodies against PNA and GM130 (a Golgi marker) in NIH3T3 cells after *O*-glycosylation inhibition (Figure 1B, panels b and e). Under normal conditions, PNA was hardly detectable in the cells (Figure 1B, panel a), whereas strong PNA immunoreactivity was detected in the cytoplasm near the nucleus 1 d after GalNAc-bn treatment (Figure 1B, panel d). The PNA staining pattern was very similar to that obtained with the anti-GM130 antibody, which recognizes the Golgi apparatus (cf. Figure 1B, panels d and e). As shown in Figure 1B panel f, the PNA immunoreactivity strikingly overlapped with GM130. Furthermore, this overlapping staining pattern was also observed in Colo 205 cells in response to GalNAc-bn treatment (Figure S1B). These findings indicate that inhibition of *O*-glycosylation by GalNAc-bn specifically occurs in the Golgi apparatus. Hereafter, the effect of GalNAc-bn on glycosylation in the Golgi apparatus will be tentatively called Golgi stress.

### Expression of the ER resident chaperone HSP47 was elevated during *O*-glycosylation inhibition (Golgi stress)

Protein expression is generally reduced when glycosylation is inhibited [24,40]. Therefore, proteins whose synthesis is increased in response to *O*-glycosylation inhibition are likely involved in Golgi protection. Therefore, we next sought to identify molecules whose expression was increased 1 d after GalNAc-bn treatment using two-dimensional polyacrylamide gel electrophoresis. We used Colo 205 cells because *O*-glycosylation is very active in these cells; therefore, *O*-glycosylation inhibition is expected to effectively and widely influence protein production in these cells.

Several spots exhibited a 0.1- to 0.9-fold decrease in signal intensity; however, 2 major spots exhibited a 1.3-fold increase in intensity. We purified one of the spots with at least a 1.3-fold increase in signal intensity, and subsequent mass spectrometric analysis strongly suggested that this protein was HSP47.

**Figure 2:**
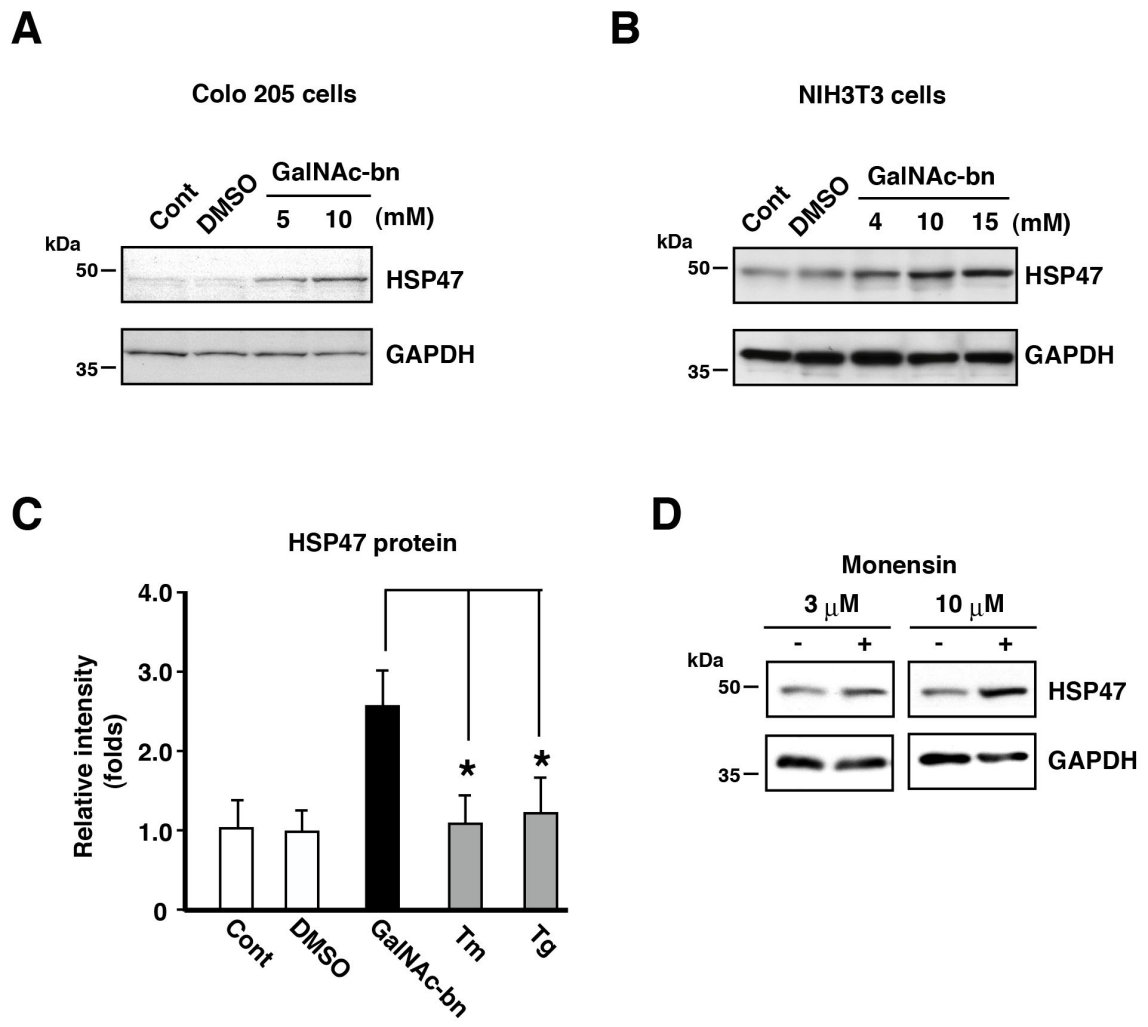
Golgi stress-induced elevation of HSP47 protein expression. (A, B) Western blotting analysis showed a remarkable and dose-dependent increase in HSP47 protein levels after GalNAc-bn stimulation (A, Colo 205 cells; B, NIH3T3 cells). (C) Quantification of the protein bands in NIH3T3 cell samples after 1-d treatment with GalNAc-bn, Tm, or Tg. Data are expressed as mean (SEM) of at least 3 independent experiments. ***p < 0.01 (Student’s *t* test). Tm, tunicamycin; Tg, thapsigargin. (D) Western blotting analysis showed a dose-dependent increase in HSP47 protein levels after monensin stimulation for 12 h in NIH3T3 cells.

Next, we examined whether HSP47 expression fluctuated after inhibition of *O*-glycosylation (Golgi stress) in Colo 205 cells and NIH3T3 cells. Immunoblotting analyses using the anti-HSP47 antibody revealed very low levels of HSP47 in cells with or without DMSO treatment (Figure 2A, B). By contrast, HSP47 protein levels increased remarkably in a dose-dependent manner after GalNAc-bn treatment in both cell lines (Figure 2A, B). Real-time PCR analysis confirmed the results from immunoblotting analyses. The expression of HSP47 mRNA was hardly detectable in cells with or without DMSO (Figure S2, Cont and DMSO). However, HSP47 mRNA levels were remarkably increased after GalNAc-bn treatment (Figure S2, Cont and GalNAc-bn). Furthermore, HSP47 mRNA and protein levels were not altered by tunicamycin(TM) or thapsigargin (Tg) treatment (Figure 2C Figure S2, Cont, Tm, and Tg), as Tm and Tg induce ER stress by preventing protein *N*-glycosylation in the ER.

We further examined whether another Golgi stressor affected the expression of HSP47. Monensin is a selective Golgi inhibitor and function by inhibiting protein transport and modification of sugar chains in the Golgi apparatus [28,41–43]. After 12 h of monensin treatment, NIH3T3 cells exhibited a remarkable dose-dependent increase in HSP47 protein levels (Figure 2D). These findings indicate that Golgi stress induced by not only GalNAc-bn but also monensin elicited an increase in HSP47 mRNA and protein expression in heavily *O*-glycosylated cells, such as Colo 205 and NIH3T3 cells.

HADHA, GM130, and calnexin were used as markers for mitochondria, Golgi apparatus, and the ER, respectively. HSP47 immunoreactivity in both untreated and GalNAc-bn-treated cells strikingly overlapped with calnexin immunoreactivity, but not with HADHA or GM130 immunoreactivity (Figure S3).

### HSP47 expression maintained the normal volume of the Golgi apparatus after *O*-glycosylation inhibition (Golgi stress)

**Figure 3:**
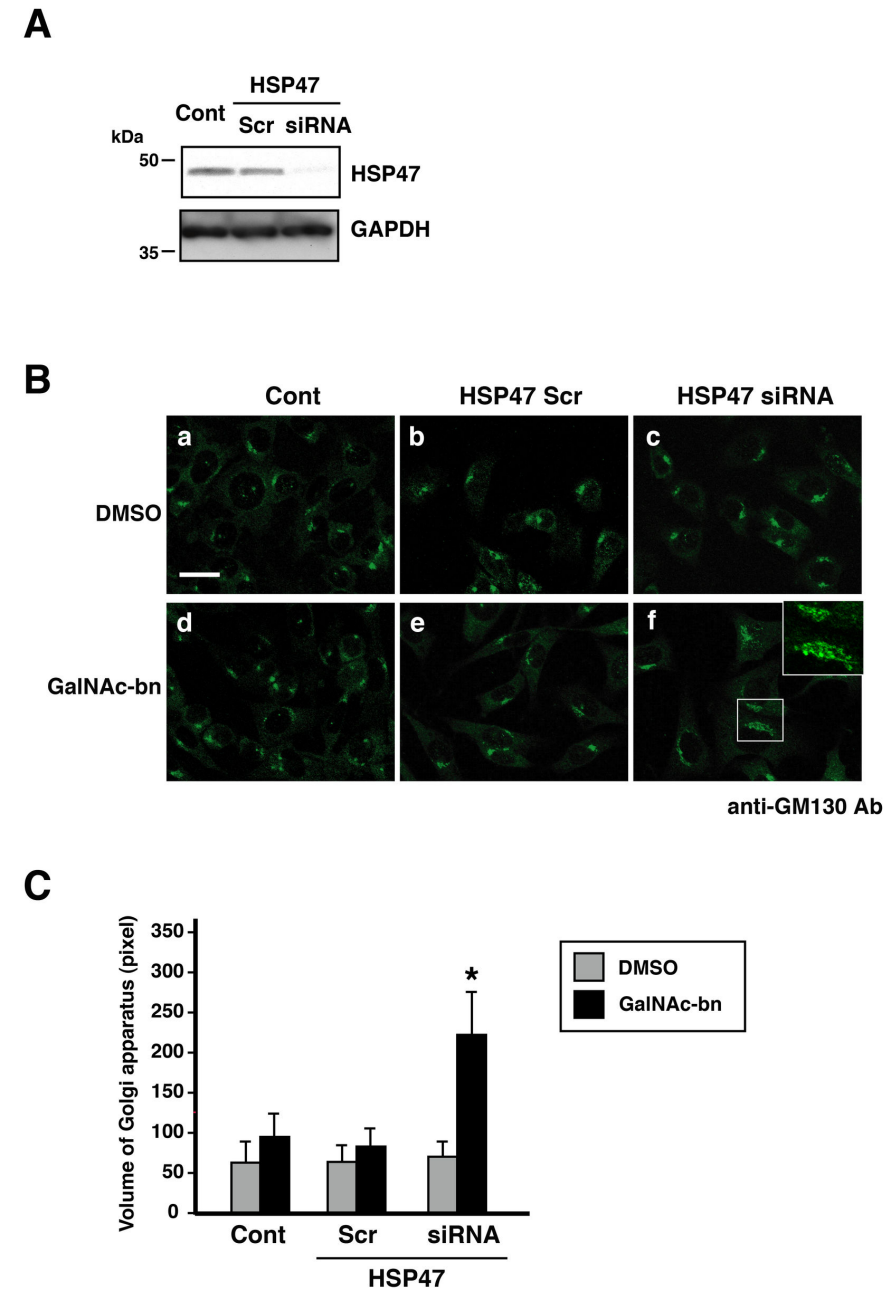
HSP47 was shown to be a regulator of Golgi volume in NIH3T3 cells. (A) Western blot analysis showed HSP47 and GAPDH protein expression 3 d after transfection with scrambled or HSP47 siRNAs. Cont, nontransfected cells; Scr, scrambled siRNA-transfected cells; siRNA, HSP47 siRNA-transfected cells. (B) NIH3T3 cells were stained with anti-GM130 antibodies with (d–f) or without (a–c) GalNAc stimulation. GalNAc-treated cells were observed at 24 h after stimulation. (f) High magnification images of a part of each photograph are shown in the white windows. Scale bar: 20 µm. (C) Results of the quantification of the volume of the Golgi apparatus in NIH3T3 cells. Golgi volume in NIH3T3 cells was measured using ImageJ software. The results are expressed as the mean (SEM) of at least 3 independent experiments. *p < 0.05 (Student’s *t* test). Cont, nontransfected cells; Scr, scrambled siRNA-transfected cells; siRNA, HSP47 siRNA-transfected cells.

As shown above, inhibition of *O*-glycosylation elevated HSP47 expression, suggesting that increased expression of HSP47 protects the Golgi apparatus during Golgi stress. Thus, to clarify the importance of HSP47 expression during the inhibition of *O*-glycosylation in NIH3T3 cells, we used the siRNA-based knockdown method to examine the effects of HSP47 depletion on NIH3T3 cell viability. To this end, we established NIH3T3 cells in which HSP47 expression was suppressed by HSP47-targeted siRNA. As shown in Figure 3A and Supplementary Figure S4, HSP47 protein expression in HSP47-targeted siRNA-transfected cells was 5% that of HSP47 expression in control and scrambled siRNA-transfected cells. Therefore, the HSP47-targeted siRNA used in this study effectively suppressed the expression of endogenous HSP47 in NIH3T3 cells.

The localization of the Golgi apparatus in untransfected control cells, scrambled siRNA-transfected cells, and HSP47 siRNA-transfected cells treated with DMSO or GalNAc-bn is shown in Figure 3B. The Golgi apparatus labeled by GM130 was localized in the perinuclear region (Figure 3B, panel a, 3C; (a) 66.21 ± 22.47 pixels), and the location of the Golgi apparatus in the scrambled siRNA- and HSP47 siRNA-transfected cells (Figure 3B, panels b and c, 3C; (b) 69.00 ± 20.08 pixels, (c) 76.82 ± 20.56 pixels) was similar to that in untransfected control cells (Figure 3B, panel a, 3C). Even after Golgi stress, the scrambled siRNA-transfected cells exhibited no significant change in the Golgi apparatus (Figure 3B, panels d and e, 3C; (d) 90.02 ± 28.29 pixels, (e) 83.60 ± 20.22 pixels), compared to control cells (Figure 3B, panel a, 3C). By contrast, HSP47 siRNA-transfected cells exhibited an increase in the volume of the Golgi apparatus after GalNAc-bn treatment (Figure 3B, panel f, 3C; (f) 217.22 ± 72.31 pixels), compared to cells transfected with HSP47 siRNA alone or GalNAc-bn-treated control cells (Figure 3B, panels c–e, 3C). Three days after GalNAc-bn treatment of HSP47 siRNA transfected cells, the Golgi apparatus in these cells consisted of spatially enlarged curved parallel arrays in the cytoplasm (Figure 3B, panel f). These findings indicate that increased HSP47 expression in the ER during Golgi stress was closely involved in maintaining the volume of the Golgi apparatus.

### Does HSP47 protect cells from Golgi stress?

**Figure 4:**
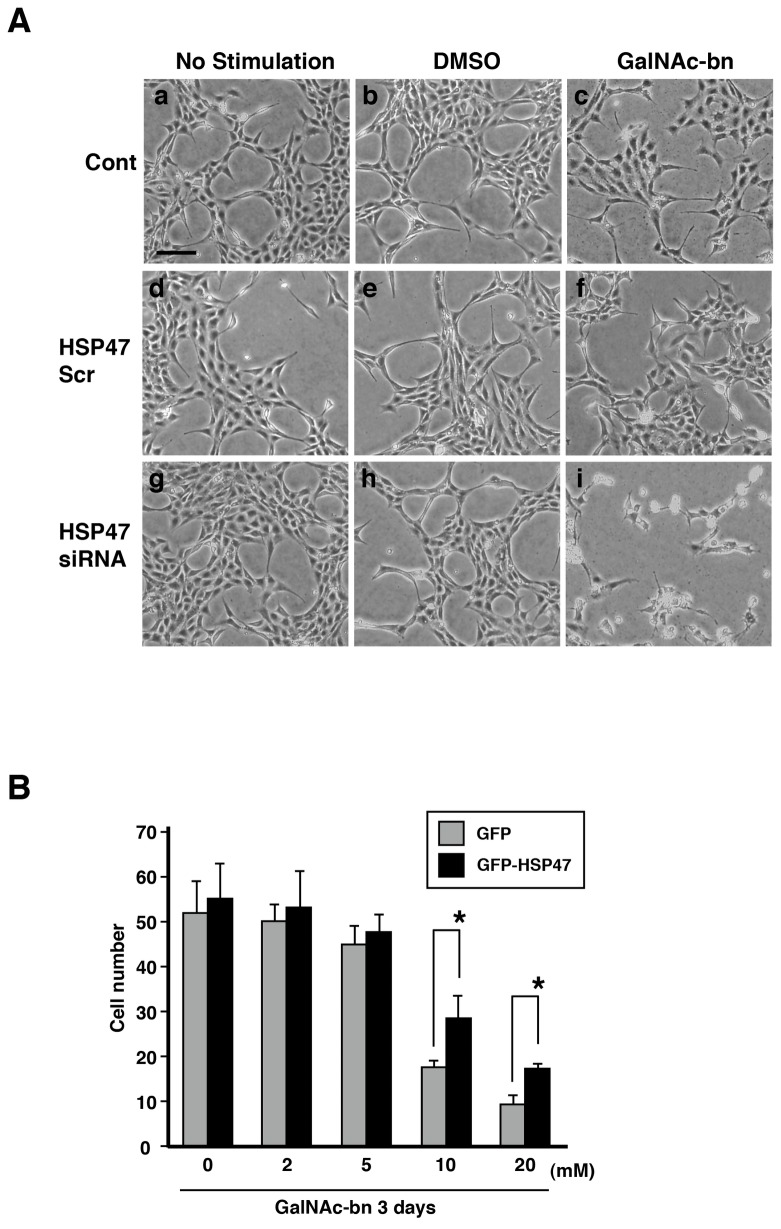
HSP47 expression protected cells from Golgi stress-induced cell death. (A) Representative phase-contrast micrographs of NIH3T3 cells. (a–c) nontransfected cells, (d–f) scrambled siRNA-transfected cells, and (g–h) HSP47 siRNA-transfected cells. GalNAc-treated cells were observed at 3 d after stimulation. Scale bar: 20 µm. (B) Viability of NIH3T3 cells 1 d after GalNAc-bn treatment (0–20 mM) as measured by a cell counting assay. Quantitative data are expressed as mean (SEM) of at least 3 independent experiments. *p <0.05 (Student’s *t* test).

We sought to determine whether the increase in the volume of the Golgi apparatus in the HSP47-knockdown cells after Golgi stress reflected hyperfunction or hypofunction. To this end, we examined the morphology of NIH3T3 cells under Golgi stress. First, we assessed the time course of the morphological changes in HSP47 siRNA-transfected cells during GalNAc–bn treatment using light microscopy. No obvious alterations in morphology were observed in untransfected control cells and scrambled siRNA-transfected cells even on day 3 after GalNAc-bn treatment (Figure 4A, panels a–f). However, when HSP47 siRNA-transfected cells were treated with GalNAc-bn, an apparent morphological change was identified 3 d after treatment (Figure 4A, panel i), although inhibition of HSP47 expression alone failed to induce the morphological change (Figure 4A, panels g and h). GalNAc-bn treatment of HSP47 siRNA-transfected cells elicited cell shrinkage or a cell death-associated morphology (Figure 4A, panel i).

We postulated that if HSP47 repression plays a key role in this morphological change following GalNAc-bn treatment, recovery of HSP47 expression in HSP47 knockdown NIH3T3 cells should prevent cell shrinkage. Thus, 2 d after HSP47 siRNA transfection, NIH3T3 cells were retransfected with empty vector control or a human HSP47 (hHSP47) construct harboring neutral mutations in the siRNA oligonucleotide sequence. Cell numbers of empty vector control and hHSP47-expressing NIH3T3 cells decreased in a dose-dependent manner after GalNAc-bn treatment. However, siRNA-transfected cells overexpressing hHSP47 exhibited an approximately 2-fold increase in cell number compared to NIH3T3 cells transfected with the empty vector control (Figure 4B). Collectively, our findings indicate that HSP47 expression may play a role in protecting NIH3T3 cells from Golgi stress-induced cell death.

**Figure 5:**
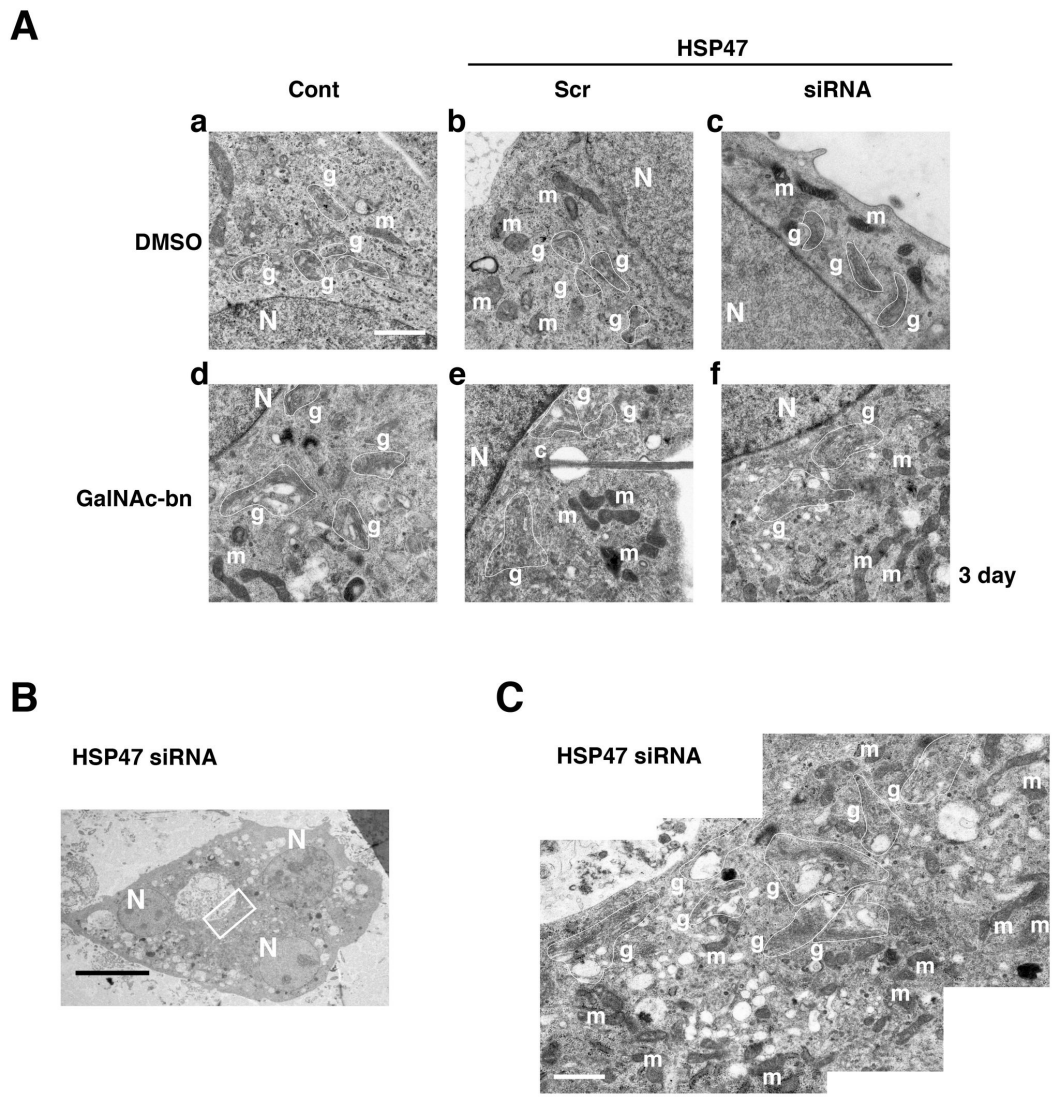
Golgi stress induced Golgi disassembly in HSP47 siRNA-transfected NIH3T3 cells. Representative electron micrographs of NIH3T3 cells. (A) Electron micrographs of NIH3T3 cells at 2 d after transfection with scrambled or HSP47 siRNAs and 3 d after treatment with DMSO or GalNAc-bn. GalNAc-bn treatment induced numerous vacuoles around the Golgi apparatus. Cont, untransfected cells; Scr, scrambled siRNA-transfected cells; siRNA, HSP47 siRNA-transfected cells. N, nucleus; g, Golgi apparatus; m, mitochondria; c, primary cilium. Scale bar: 4 µm. (B) Electron micrographs of NIH3T3 cells at 2 d after transfection with HSP47 siRNA and 3 d after treatment with GalNAc-bn. There are 3 nuclei in 1 cell and numerous vacuoles extending into the cytoplasm. N, nucleus. Scale bar: 20 µm. (C) Selection of high magnification images of the small rectangles in panel C. Numerous vacuoles were observed not only around the Golgi apparatus but also in the cytoplasm. g, Golgi apparatus; m, mitochondria. Scale bar: 4 µm.

Without GalNAc-bn treatment, the ultrastructure of the Golgi apparatus was similar in control cells with or without DMSO treatment (1 d or 3 d), untransfected control cells, scrambled siRNA-transfected cells, and HSP47 siRNA-transfected cells (Figure 5A Figure S5, panels a–c). The Golgi apparatus in these cells consisted of curved parallel arrays of flattened cisterns expanding at their lateral ends. These arrays were rather compactly located in the perikarya, and a number of small vesicles were associated with these arrays. One day after GalNAc-bn treatment, a small number of vacuoles appeared around the Golgi apparatus in untransfected control cells, scrambled siRNA-transfected cells, and HSP47 siRNA-transfected cells (Figure S5, panels d–f). Three days after GalNAc-bn treatment, a small number of vacuoles were still observed around the Golgi apparatus in untransfected control cells and scrambled siRNA-transfected cells (Figure 5A, panels d and e). By contrast, HSP47 siRNA-transfected cells exhibited a marked increase in the number of vacuoles, many of which were located around the Golgi apparatus, at 3 d after GalNAc-bn treatment (Figure 5A, panel f; Figure 5B, C). In addition, in some cells, these vacuoles dispersed into the cytoplasm (Figure 5B, C), and in a few cases, most of the cytoplasm was occupied by the vacuoles. GalNAc-bn treatment of siRNA HSP47-transfected cells caused an evident change in the Golgi apparatus after 3 d. The curved parallel arrays were disassembled and dispersed more widely in the perikarya of these cells than in control cells (Figure 5C). Many of the disassembled curved parallel arrays were associated with areas close to the inner and outer surfaces of the arrays, a part of the disassembled curved parallel arrays in the cytoplasm between different parallel arrays. Furthermore, consistent with light microscopic observations, a few cells exhibited split nuclei with condensed chromatin (Figure 5B). However, formation of apoptotic bodies was not observed. Therefore, the increase in Golgi volume observed at the light microscopic level after Golgi stress may be attributed to the disassembly of the Golgi apparatus.

### HSP47 expression protected cells from Golgi stress-induced apoptosis

As described above, GalNAc-bn treatment of HSP47 knockdown cells induced (i) cell shrinkage; (ii) disassembly of the Golgi apparatus; (iii) appearance of a number of large vacuoles in the cytoplasm, particularly near the Golgi apparatus; and (iv) splitting of the nucleus with chromatin condensation, suggesting that cell death was in progress in these cells [44,45]. Therefore, the TUNEL assay was performed and the enzymatic activities of various caspases were measured to assess cell death.

**Figure 6:**
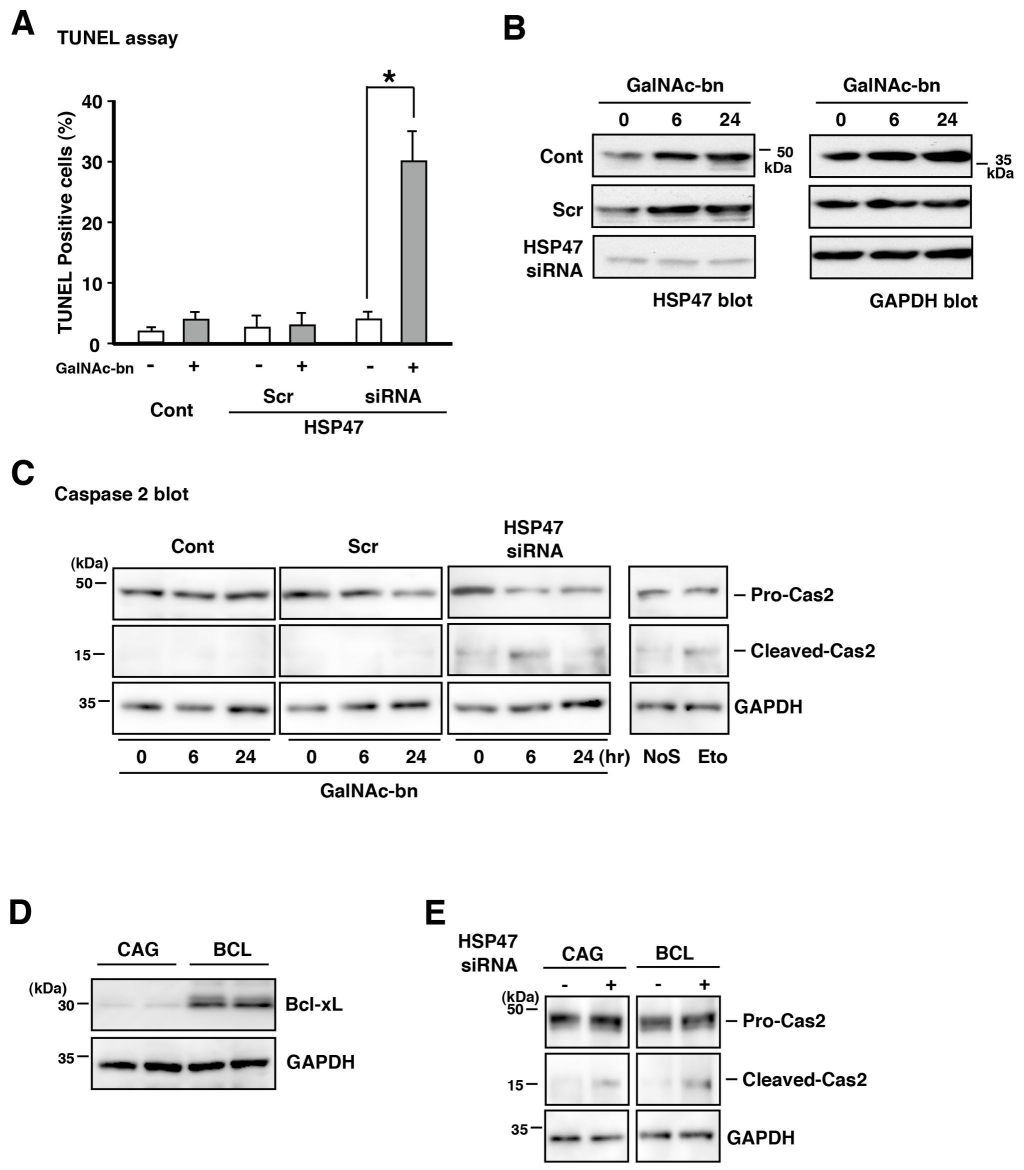
HSP47 expression protected NIH3T3 cells from Golgi stress-induced apoptosis. (A) TUNEL-positive cells were significantly increased in HSP47 siRNA-transfected NIH3T3 cells after GalNAc-bn treatment. Results are presented as the percentage of TUNEL-positive cells over total cells, normalized to untreated controls. Data are expressed as the mean (SEM) of at least 3 independent experiments. *p < 0.05 (Student’s *t* test). (B) The expression of HSP47 or GAPDH protein in NIH3T3 cells after treatment with GalNAc-bn for the indicated periods was examined by western blot analysis using anti-HSP47 or anti-GAPDH antibodies. (C) NIH3T3 cells treated with GalNAc-bn for the indicated periods were examined by western blot analysis using antibody against caspase-2. NIH3T3 cells treated with etoposide (Eto) were used as a positive control for caspase-2 cleavage induction. Cont, untransfected cells; Scr, scrambled siRNA-transfected cells; siRNA, HSP47 siRNA-transfected cells, NoS, no stimulated cells. (D) Western blot analysis showed a remarkable increase in Bcl-xL protein levels in the NIH3T3 cell line stably over-expressing Bcl-xL (BCL) in comparison with NIH3T3 cells expressing the pCAGGS empty vector (CAG). (E) CAG cells and BCL cells treated with GalNAc-bn for 6 h were examined by western blot analysis using an antibody against caspase-2 after HSP47 siRNA transfection.

Assessment of cell death using TUNEL staining revealed that less than 4% of cells in the untransfected control and scrambled siRNA-transfected cells were killed by treatment with GalNAc-bn for 3 d (Figure 6A). By contrast, more than 30% of the HSP47 siRNA-transfected cells died after GalNac-bn treatment (Figure 6A).

In addition, we confirmed that 6 h and 24 h GalNAc-bn treatments upregulated HSP47 expression levels in only the untransfected control and scrambled siRNA-transfected cells (Figure 6B). As shown in Figure 6C, a 6 h GalNAc-bn treatment elicited higher levels of cleaved caspase-2, which belongs to the initiator caspase family [46], in the HSP47 siRNA-transfected cells than in the untransfected control and scrambled siRNA-transfected cells.

We further investigated whether caspase-2 cleavage was initially induced by this Golgi stress caused by the GalNAc-bn treatment with inhibition of HSP47 expression. We made a NIH3T3 cell line stably expressing Bcl-xL (Figure 6D). It has been reported that cells over-expressing Bcl-xL are highly resistant to mitochondrial-dependent cell death [47,48]. GalNAc-bn treatment with inhibition of HSP47 expression in CAG cells that expressed the pCAGGS empty vector resulted in cleavage of caspase-2 (Figure 6E, CAG column). Furthermore, caspase-2 cleavage was also observed in BCL cells that over-expressed Bcl-xL after GalNAc-bn treatment (Figure 6E, BCL column). These results indicate that caspase-2 cleavage was initially induced by the GalNAc-bn treatment with inhibition of HSP47 expression.

**Figure 7:**
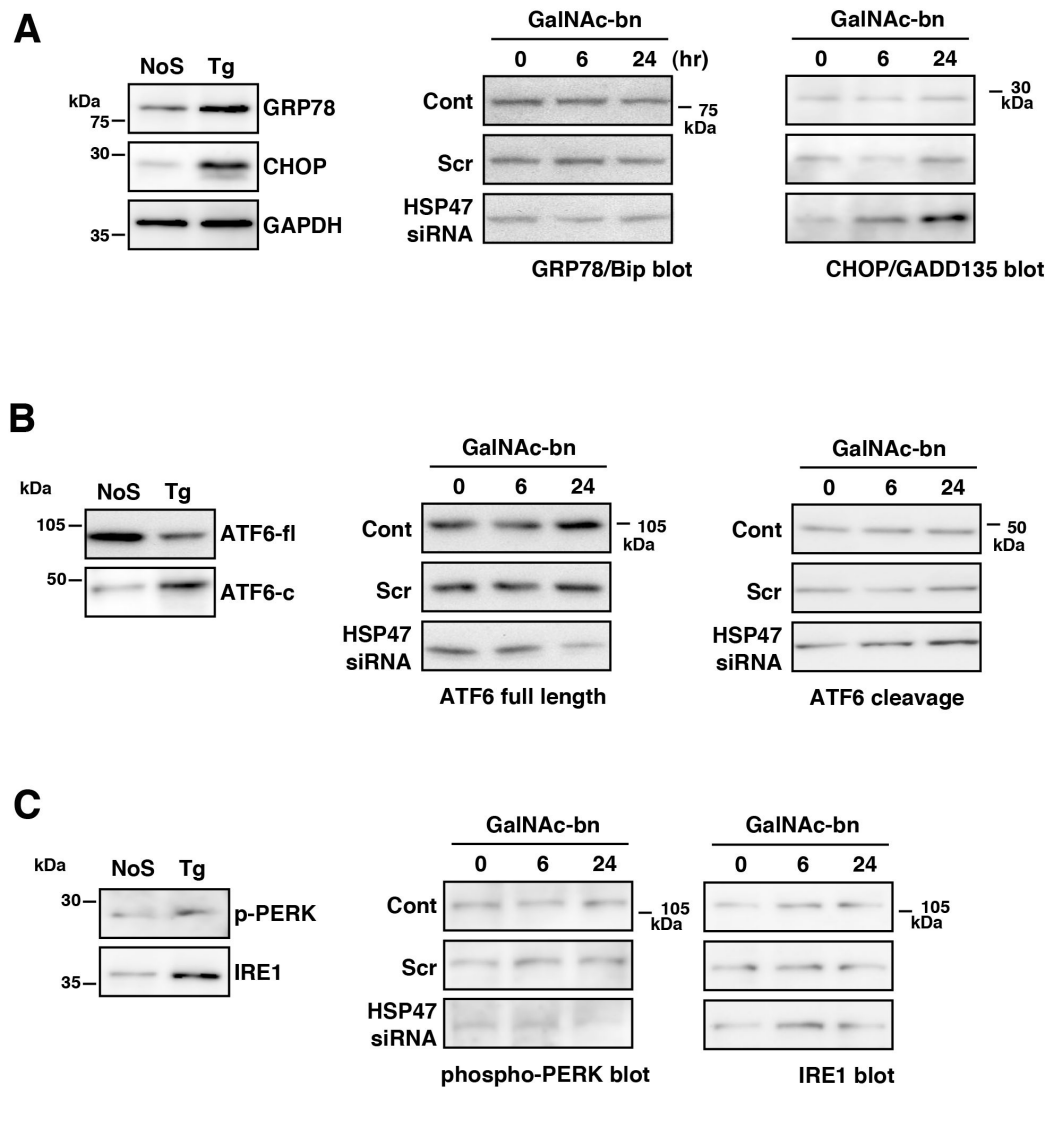
HSP47 expression affected ER-resident chaperone interactions. The expression of GRP78/Bip, CHOP/GADD135, full-length ATF6, cleaved ATF6, phospho-PERK, or IRE1 protein in NIH3T3 cells after treatment with GalNAc-bn for the indicated periods was examined by western blot analysis using anti-GRP78/Bip antibodies (A), anti-GADD153 (CHOP) antibodies (A), anti-ATF6 antibodies (B), anti-phosphoPERK antibodies (C), or anti-IRE1α antibodies (C). NIH3T3 cells treated with thapsigargin (Tg) were used as positive controls for these UPR-related molecule activation. Cont, untransfected cells; Scr, scrambled siRNA-transfected cells; siRNA, HSP47 siRNA-transfected cells; NoS, no stimulated cells; ATF6-fl, ATF6 full length; ATF6-c, ATF6 cleavage.

Furthermore, HSP47 expression was observed in the ER of NIH3T3 cells during Golgi stress (Figure S3). We next examined the expression of ER-resident chaperones, such as GRP78/Bip, which facilitate protein folding and CHOP/GADD135 expression *via* the PERK-ATF4 pathway [49,50]. Cell survival or cell death depends on the balance between CHOP/GADD135 and GRP78/Bip expression. Therefore, we examined the expression of CHOP/GADD135 and GRP78/Bip in untransfected control, scrambled siRNA-transfected, and HSP47 siRNA-transfected cells under Golgi stress. GRP78/Bip expression in these cells exhibited no increase after treatment with GalNAc-bn for 6 h and 24 h (Figure 7A, GRP78/Bip columns). However, the HSP47 siRNA-transfected cells alone exhibited decreased GRP78/Bip expression in comparison with untransfected control or scrambled siRNA-transfected cells during Golgi stress (Figure 7A, GRP78/Bip columns). Furthermore, an increase in CHOP/GADD135 expression was observed in the HSP47 siRNA-transfected cells only after treatment with GalNAc-bn for 6 h and 24 h (Figure 7A, CHOP/GADD135 columns).

We further investigated the expression or activation of unfolded protein response (UPR)-related molecules, i.e., ATF6, a sensor-transcription factor embedded in the ER, PERK, a sensor-kinase located in the ER membrane, and IRE1α, a sensor RNase located in the ER membrane [51]. We examined full-length ATF6 expression and cleavage levels by western blotting (Figure 7B) and levels of IRE1 expression and PERK phosphorylation (Figure 7C). Full-length ATF6 expression did not increase in these cells after treatment with GalNAc-bn for 6 h and 24 h (Figure 7B, ATF6 full length columns). However, the HSP47 siRNA-transfected cells alone exhibited decreased full-length ATF6 expression in comparison with untransfected control or scrambled siRNA-transfected cells during Golgi stress (Figure 7B, ATF6 full length columns). Furthermore, an increase in cleaved ATF6 expression was observed in the HSP47 siRNA-transfected cells only after treatment with GalNAc-bn for 6 h and 24 h (Figure 7B, ATF6 cleavage columns).

PERK activation levels decreased in the HSP47 knockdown cells compared to that of untransfected control and scrambled siRNA-transfected cells after treatment with GalNAc-bn (Figure 7C, phospho-PERK columns). On the other hand, HSP47 expression levels did not influence IRE1 expression following GalNAc-bn treatment (Figure 7C, IRE1 columns). These findings indicate that Golgi stress influences not only the Golgi apparatus but also the ER, as part of the ER stress response *via* UPR pathways.

### Golgi stress and HSP47 inhibition together induced cytochrome *c* efflux

**Figure 8:**
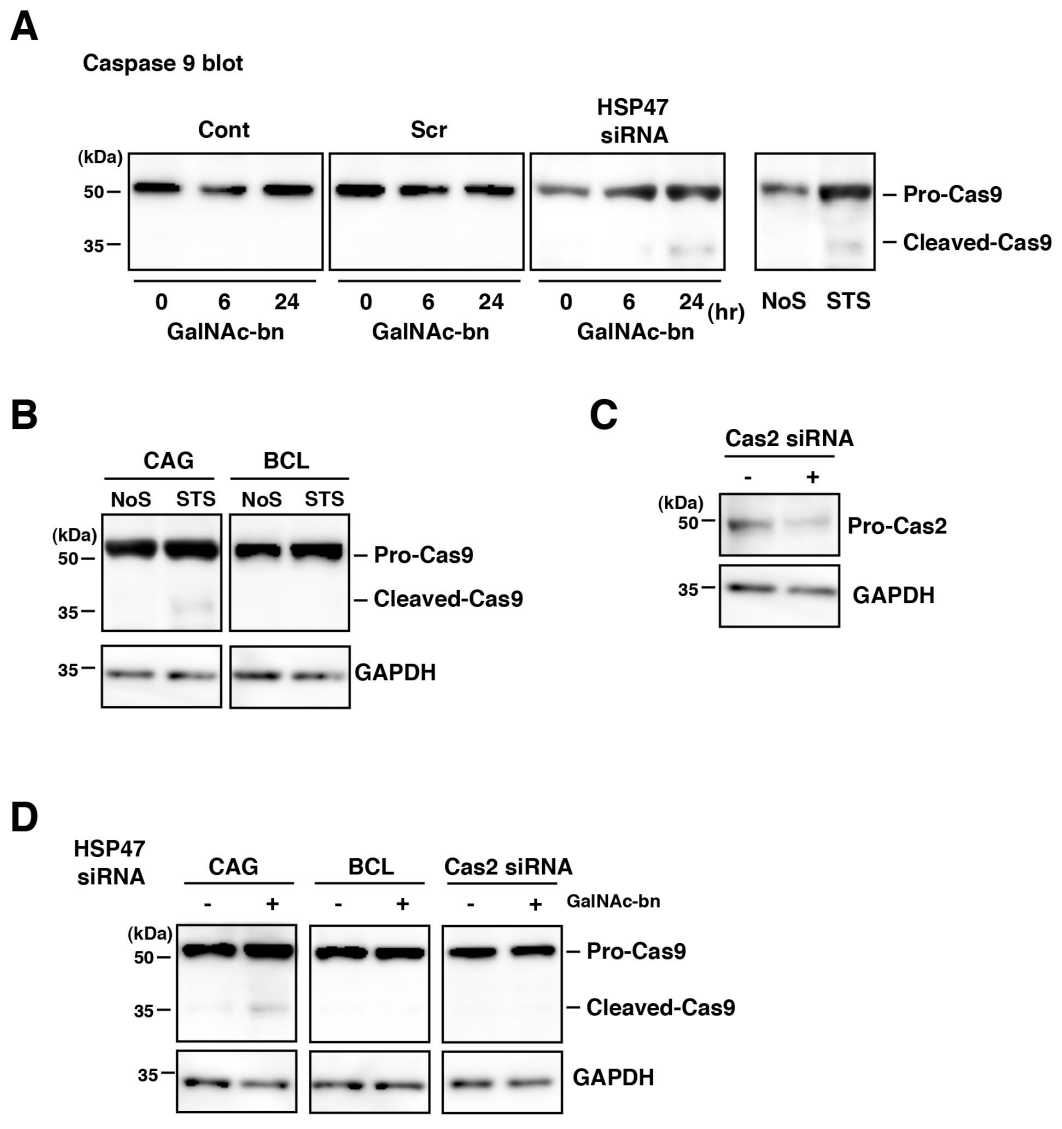
HSP47 expression affected mitochondria caspase-9 cleavage. (A) NIH3T3 cells treated with GalNAc-bn for the indicated periods were examined by western blot analysis using an antibody against caspase-9. NIH3T3 cells treated with staurosporine (STS) were used as a positive control for caspase-9 cleavage induction. Cont, untransfected cells; Scr, scrambled siRNA-transfected cells; siRNA, HSP47 siRNA-transfected cells; NoS, no stimulated cells. (B) CAG and BCL cells treated with STS for 12 h were examined by western blot analysis using an antibody against caspase-9. (C) Western blot analysis showed caspase-2 and GAPDH protein expression 2 d after transfection with caspase-2 siRNA. (D) CAG, BCL, and caspase-2 siRNA transfected NIH3T3 (Cas2 siRNA) cells treated with GalNAc-bn for 24 h were examined by western blot analysis using an antibody against caspase-9 after HSP47 siRNA transfection.

We further investigated the activation of caspase-9 by the Golgi stress induced by GalNAc-bn treatment with inhibition of HSP47 expression. Cleaved caspase-9 was not detected after GalNAc-bn treatment in untransfected control and scrambled siRNA-transfected cells. Furthermore, increases in the levels of cleaved caspase-9 were not be detected with treatment of GalNAc-bn for 0 h and 6 h (Figure 8A). Subsequently, treatment with GalNAc-bn for 24 h elicited caspase-9 cleavage in the HSP47 knockdown cells (Figure 8A, HSP47 siRNA 24 h columns), which strongly suggests that Golgi stress caused by the GalNAc-bn treatment with inhibition of HSP47 expression influenced both ER and mitochondrial functions because activation of UPR and caspase-9 strongly affect the ER [8] and mitochondria [52,53], respectively.

Thus, to clarify the importance of caspase-2 cleavage during Golgi stress in NIH3T3 cells, we used caspase-2 siRNA to examine the cleavage levels of caspase-9 after GalNAc-bn treatment of HSP47-depleted NIH3T3 cells. As shown in Figure 8C, caspase-2 protein expression was suppressed effectively by caspase-2-targeted siRNA treatment in NIH3T3 cells. Cells over-expressing Bcl-xL (BCL) are highly resistant to mitochondrial-dependent cell death. Thus, BCL cells were highly resistant to caspase-9 cleavage with induction of mitochondrial stress following staurosporine (STS) treatment (Figure 8B). GalNAc-bn treatment with inhibition of HSP47 expression of CAG cells elicited cleaved caspase-9 (Figure 8D, CAG column). However, cleavage of caspase-9 was not observed in BCL cells or in caspase-2-depleted cells with inhibition of HSP47 expression (Figure 8D, BCL and Cas2 siRNA columns). These results indicate that caspase-2 cleavage was required for caspase-9 cleavage by Golgi stress in HSP47-depleted NIH3T3 cells.

**Figure 9:**
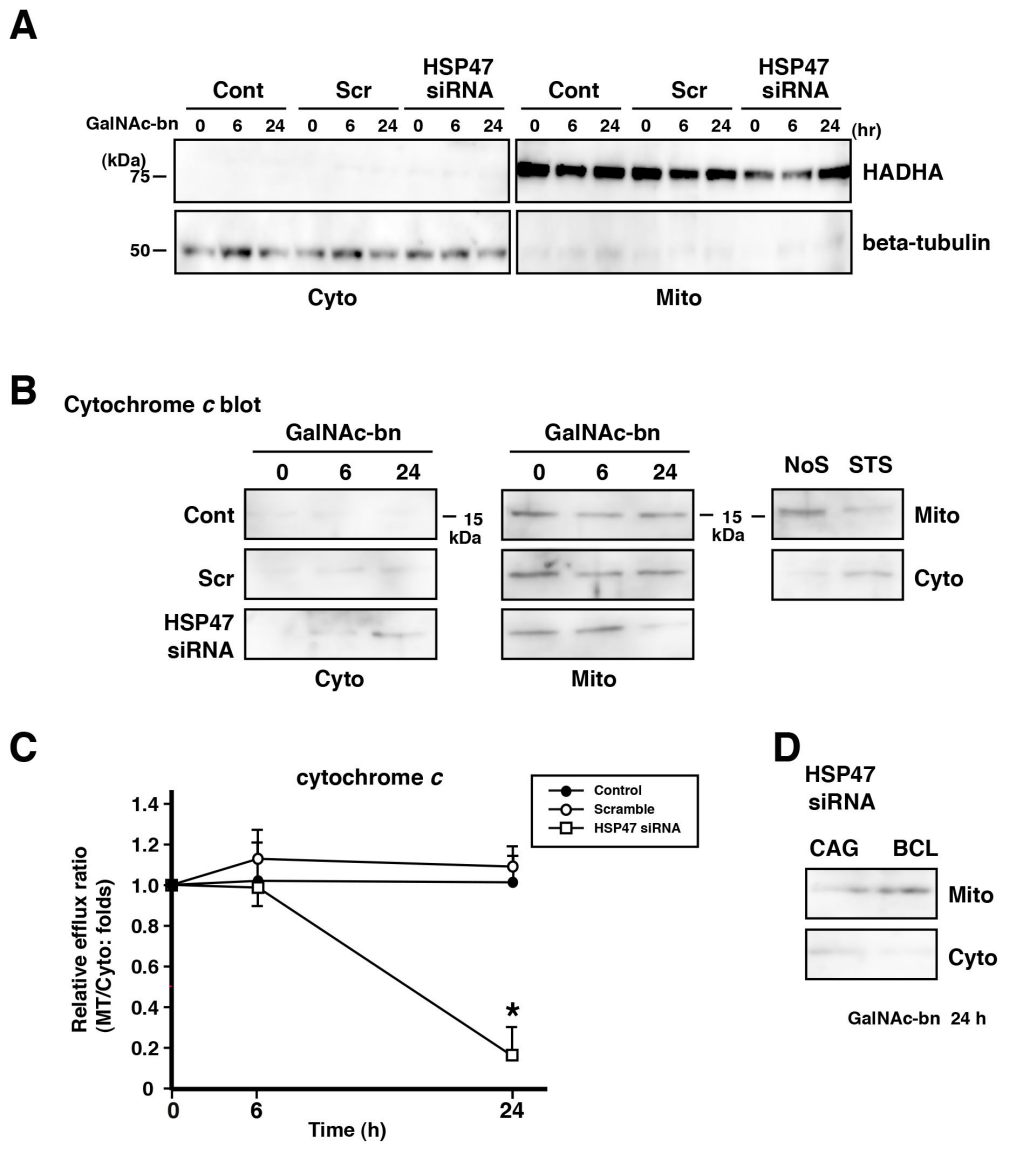
HSP47 expression affected mitochondria cytochrome *c* efflux. (A) NIH3T3 cells were fractionated into cytoplasmic (Cyto) and mitochondrial (Mito) fractions, which were analyzed by western blotting using antibodies against HADHA (mitochondrial marker) and β-tubulin (cytoplasmic marker). (B) Western blot analysis showed cytochrome *c* protein levels in each of the Cyto and Mito fractions for 2 d after transfection with scrambled or HSP47 siRNAs of NIH3T3 cells treated with GalNAc-bn for the indicated periods. Cont, nontransfected cells; Scr, scrambled siRNA-transfected cells; siRNA, HSP47 siRNA-transfected cells. (C) Quantification of the intensity of protein bands from GalNAc-bn-treated samples of (B). Cytochrome *c* release from the mitochondrial to the cytoplasmic fraction in HSP47 siRNA-transfected NIH3T3 cells at 24 h after GalNAc-bn stimulation. Data are presented as the relative efflux ratio (Mito/Cyto) compared to that of the corresponding untreated control group, which was set at 1.0. Cont, untransfected cells; Scr, scrambled siRNA-transfected cells; siRNA, HSP47 siRNA-transfected cells. (D) Western blot analysis showed cytochrome c protein levels of the Cyto and Mito fractions for 2 d after transfection with HSP47 siRNAs of CAG and BCL cells at 24 h after GalNAc-bn stimulation.

As described above, Golgi stress caused by the inhibition of HSP47 expression elicited caspase-9 activation. Given that caspase-9 is activated by mitochondrial dysfunction [48,52,53], we examined whether Golgi stress in the absence of HSP47 expression provoked cytochrome *c* efflux from the mitochondria to the cytoplasm by western blot analysis of cytoplasmic (Cyto) and mitochondrial (Mito) fractions. We first confirmed the cellular fractionation by HADHA (mitochondria-specific protein) and β-tubulin (cytoplasm-specific protein) blotting as internal controls (Figure 9A). In the absence of HSP47 expression, cytochrome *c* was detected in the cytoplasmic fraction of the cells at 24 h after Golgi stress (Figure 9B, C). However, cytoplasmic cytochrome *c* was not observed in BCL cells with inhibition of HSP47 expression at 24 h after Golgi stress (Figure 9D).

### Golgi stress had no role in collagen dynamics during progression of the cell-death signal

**Figure 10:**
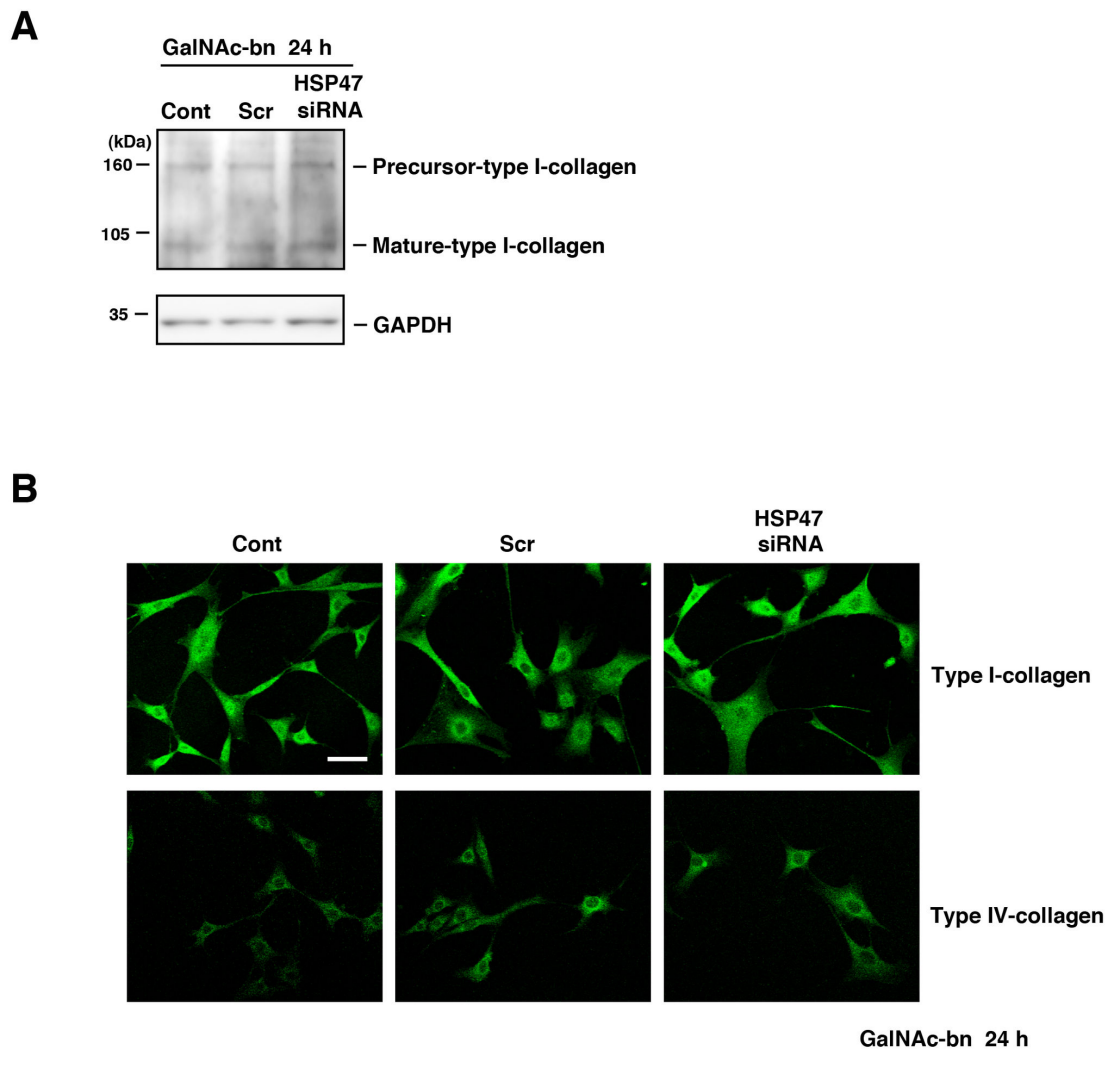
Collagen dynamics were not involved in Golgi stress signaling. (A) NIH3T3 cells treated with GalNAc-bn for the indicated periods were examined by western blot analysis using antibodies against type I collagen. (B) NIH3T3 cells were stained with anti-type I collagen antibodies and anti-type IV collagen antibodies at 24 h after GalNAc-bn stimulation. Cont, untransfected cells; Scr, scrambled siRNA-transfected cells; siRNA, HSP47 siRNA-transfected cells. Scale bar: 50 µm.

HSP47 is a well-known collagen-specific molecular chaperone [54,55]. A previous report discussing the relationship between collagen expression levels and cell death indicated that type I collagen degradation via the ERK pathway promotes cell death [56]. We first examined the levels of type I collagen expression during the progression of cell death signaling using western blots. Type I collagen levels did not increase in the HSP47 knockdown cells compared to that of untransfected control and scrambled siRNA-transfected cells (Figure 10A). We further performed immunostaining using an antibody against type I collagen in NIH3T3 cells after *O*-glycosylation inhibition. There was no apparent change in the HSP47 siRNA-transfected cells after GalNAc-bn treatment, compared to that of control cells 24 h after Golgi stress (Figure 10B; Type I-collagen).

Furthermore, previous reports have indicated accumulation of type IV collagen in ER-induced apoptosis in HSP47-knockout mice [57]. Thus, to investigate the effect of GalNAc-bn treatment on the induction of collagen on the ER in HSP47-knockdown NIH3T3 cells during the progress of the cell death signal, we performed immunostaining with antibodies against type IV collagen in NIH3T3 cells after O-glycosylation inhibition. However, no apparent change in type IV collagen localization was seen in the HSP47 siRNA-transfected cells after GalNAc-bn treatment, compared to that of control cells during 24 h after Golgi stress (Figure 10B; Type IV-collagen). These findings indicate that collagen dynamics do not play a role in the progression of cell death due to Golgi stress.

## Discussion

The present study demonstrated that Golgi stress caused by the inhibition of *O*-glycosylation induced the expression of HSP47 in the ER of NIH3T3 cells and that downregulation of HSP47 caused Golgi dysfunction, leading to cell death (Figure S6).

### Inhibition of *O*-glycosylation (Golgi stress) upregulated the ER-resident chaperone HSP47

As shown in this study, Golgi stress upregulated the ER-resident chaperone HSP47. At present, the molecular mechanism underlying the elevation of HSP47 in response to *O*-glycosylation inhibition is unknown. HSP47 is a collagen-specific, ER-resident molecular chaperone that interacts with pro-collagen during collagen formation [58,59]. It has also been suggested that HSP47 is involved in the transport of pro-collagen from the ER to the Golgi apparatus [60,61]. Pro-collagen is dissociated from HSP47 in the *cis*-Golgi or the ER-Golgi intermediate compartment areas to enter the Golgi apparatus. Because HSP47 contains an ER retention signal, the dissociated HSP47 returns to the ER [58]. Given that *O*-glycosylation is associated with the maturation of pro-collagen [62], it may be postulated that inhibition of *O*-glycosylation in the Golgi apparatus influences HSP47 synthesis.

### Inhibition of HSP47 expression during Golgi stress caused cell death due to Golgi dysfunction

It has been well established that perturbation of mitochondrial function is involved in apoptosis, and many studies have reported apoptosis-related molecules around the mitochondria [17,18,48–50,63]. Apoptosis-inducing stimuli elicit the release of mitochondrial cytochrome *c*, which binds to Apaf-1 to activate caspase-9, one of the initiator caspases with a long pro-domain. Activated caspase-9 then cleaves and activates effector caspases, including caspase-3 and caspase-7, which have relatively short pro-domains.

Recently, several investigators, including our group, have established that neuronal death in Alzheimer’s disease (AD), Parkinson disease, Huntington disease, amyotrophic lateral sclerosis, and cerebral ischemia is attributable to ER stress caused by ER dysfunction [12,64–67]. For example, familial AD-linked presenilin 1 (PS1) mutations increase the vulnerability to ER stress by altering the UPR [12]. In addition, caspase-1 has recently been shown to be involved in signaling pathways specific to ER stress-induced apoptosis [68,69].

However, there have been no reports showing that cell death is induced by specific Golgi stress. We have shown that inhibition of *O*-glycosylation elevates the expression of HSP47, which is predominantly localized in the ER. Furthermore, consistent with previous studies, inhibition of *O*-glycosylation (specific Golgi stress) alone did not induce cell death (Figure 4). However, when HSP47 expression was downregulated, inhibition of *O*-glycosylation elicited Golgi disassembly, the appearance of vacuoles, splitting of nuclei (Figure 5), and an increase in TUNEL-positive cells (Figure 6), showing that downregulation of the ER-resident chaperone HSP47 and Golgi stress induced cell death.

Caspase-2 is localized in the Golgi apparatus and may be important for unlocking the role of the Golgi complex in apoptotic signaling [1,70]. In HSP47 knockdown cells, cleaved forms of caspase-2 were detected under basal conditions. Moreover, inhibition of HSP47 expression during Golgi stress increased the cleavage of caspase-2 (Figure 6). These findings, together with the morphological change in the Golgi apparatus mentioned above and the increase in the number of TUNEL-positive cells, point to the mechanism underlying the Golgi dysfunction caused by Golgi stress in response to the inhibition of HSP47 expression.

The molecular mechanism underlying cell death caused by Golgi stress and the inhibition of HSP47 expression is quite obscure. Previous studies have shown that the Golgi apparatus eliminates misfolded proteins that escape from the ER [71,72]. HSP47 may suppress the escape of ER proteins. To eliminate misfolded proteins, the Golgi apparatus may have stress transducers that are involved in post-ER protein quality control. When these hypothetical stress transducers located in the Golgi apparatus sense the accumulation of low quality protein produced because of Golgi stress, they may activate the Golgi-resident caspase-2.

### Golgi dysfunction caused by Golgi stress from the inhibition of HSP47 expression extended to the ER and mitochondria

Although cleavage of caspase-9 was not affected by HSP47 knockdown alone, HSP47 knockdown together with Golgi stress increased the cleavage of caspase-9, in addition to caspase-2 (Figures 6, 8). In addition, efflux of cytochrome *c* from the mitochondria to the cytoplasm was detected 24 h after the same stimulation. These findings indicate that cytochrome *c* flowed out of the mitochondria into the cytoplasm and activated caspase-9, although the possibility that activated UPR-related molecules directly activated caspase-9 cannot be ruled out [73]. In any case, it is certain that Golgi dysfunction caused dysfunction of the mitochondria and ER.

Golgi stress alone did not cause cell death (Figure 4). However, as shown in this study, Golgi stress upregulated the expression of the ER-resident HSP47. Moreover, when expression of HSP47 was inhibited, Golgi stress caused cell death and activation of the Golgi-resident caspase-2, ER-resident UPR-related molecules, and mitochondria-resident caspase-9, along with efflux of cytochrome *c* from the mitochondria to the cytoplasm (Figure S6). UPR-related molecules mediate apoptosis specifically in response to ER stress in rodents and humans [8,47].

Thus, although HSP47 is known to play a key role in protecting the Golgi apparatus from Golgi stress, the molecular cascade of this system remains unknown. Further, the molecular pathway that transduces the Golgi stress to the mitochondria is also unknown. It has been reported that the disialoganglioside GD3 is one of the mediators between the Golgi apparatus and the mitochondria [74–77]. GD3 plays an important role as an intracellular mediator of the apoptotic program in several cell types, and it is likely required for stimulating the trans-Golgi network (TGN) to protect the mitochondrial membrane [78–81]. It is well known that mitochondria are associated with the progression of apoptosis *via* the loss of mitochondrial membrane potential [81–83]. GD3 specifically induces gradual depolarization of the inner mitochondrial membrane, which is otherwise suppressed by cyclosporine A, a mitochondrial pore-opening inhibitor [81]. Furthermore, GD3 accumulation is caused by GD3 synthase and ST8 activation. ST8 is a Golgi-resident protein, and ST8 overexpression induces GD3 accumulation, mitochondrial damage, and apoptosis [84]. To protect against GD3-induced apoptosis, calnexin, one of the molecular chaperones in the ER, binds to ST8 and prevents its localization in the Golgi apparatus [77]. These results and those from our study, suggest that not only GD3 but also HSP47 is possibly associated with membrane regulation in the mitochondria, ER, and the Golgi apparatus.

The present study showed that HSP47 in the ER plays a key role in preventing Golgi dysfunction-induced cell death (Figure S6). Elucidating the novel functional roles of HSP47 remains a goal of future research.

## Supporting Information

Figure S1 GalNAc-bn treatment decreases O-glycosylation in Colo 205 cells. (A) Western blot analysis of the PNA lectin binding levels (a marker for the inhibition of O-glycosylation levels) in Colo 205 cells. Cells were treated with GalNAc-bn for 1 d. (B) Immunoreactivity of PNA lectin overlapped with the localization of the Golgi apparatus in the presence of GalNAc-bn treatment. GalNAc-bn-treated cells were observed 24 h after stimulation. Scale bar: 30 µm.
(TIF)

Figure S2 Golgi stress induced elevation of HSP47 mRNA expression levels. (A, B) Real-time PCR analysis showed increasing HSP47 mRNA levels in Colo 205 cells (A) and NIH3T3 cells (B) specifically after GalNAc-bn, and HSP47 mRNA expressions did not change after Tm, or Tg stimulation. Tm, tunicamycin; Tg, thapsigargin. Data are expressed as the mean ± SEM of at least 3 independent experiments. *p <0.05 (Student’s t test).
(TIF)

Figure S3 Intracellular localization of HSP47 in NIH3T3 cells. NIH3T3 cells were stained with anti-HSP47 antibodies and anti-HADHA antibodies (mitochondria) (A), anti-GM130 antibodies (Golgi apparatus) (B), and anti-calnexin antibodies (ER) (C) with (d–f) or without (a–c) GalNAc-bn stimulation. GalNAc-bn-treated cells were observed 24 h after stimulation. Scale bar: 20 µm.
(TIF)

Figure S4 HSP47 protein expression check by immunocytochemistry. NIH3T3 cells were stained with anti-HSP47 antibodies with (d–f) or without (a–c) GalNAc stimulation. GalNAc-treated cells were observed 24 h after stimulation. Cont, nontransfected cells; Scr, scrambled siRNA-transfected cells; siRNA, HSP47 siRNA-transfected cells. Scale bar: 20 µm.
(TIF)

Figure S5 Golgi stress induces the disassembly of the Golgi apparatus in HSP47 siRNA-transfected NIH3T3 cells. Electron micrographs of NIH3T3 cells 2 d after transfection with scrambled or HSP47 siRNAs and 1 d after treatment with DMSO or GalNAc. GalNAc treatment induced numerous vacuoles around the Golgi apparatus. Cont, untransfected cells; Scr, scrambled siRNA-transfected cells; siRNA, HSP47 siRNA-transfected cells. N, nucleus; g, Golgi apparatus; m, mitochondria; c, primary cilium. Scale bar: 4 µm.
(TIF)

Figure S6 Hypothetical pathways by which Golgi stress induces cell death of NIH3T3 cells. Golgi stress promotes ER-resident chaperone HSP47 expression and protects caspase-2 cleavage. HSP47-knockdown NIH3T3 cells exhibited increased cleavage of Golgi-resident caspase-2. Furthermore, HSP47-knockdown cells exhibited activation of ER-resident unfolded protein response (UPR)-related molecules, and efflux of cytochrome c from the mitochondria to the cytoplasm and activation of mitochondrial caspase-9. Golgi stress influences not only Golgi apparatus function but also ER and mitochondria functions and induced cell death via inhibition of the HSP47.
(TIF)

File S1 **Extended materials and methods.**
(DOCX)
