# Supplementary figures and images for: The Endoplasmic Reticulum-Resident Chaperone Heat Shock Protein 47 Protects the Golgi Apparatus from the Effects of O-Glycosylation Inhibition
Source: PLoS One. 2013 Jul 29;8(7):e69732. doi: 10.1371/journal.pone.0069732 (PMC3726774; doi:10.1371/journal.pone.0069732)

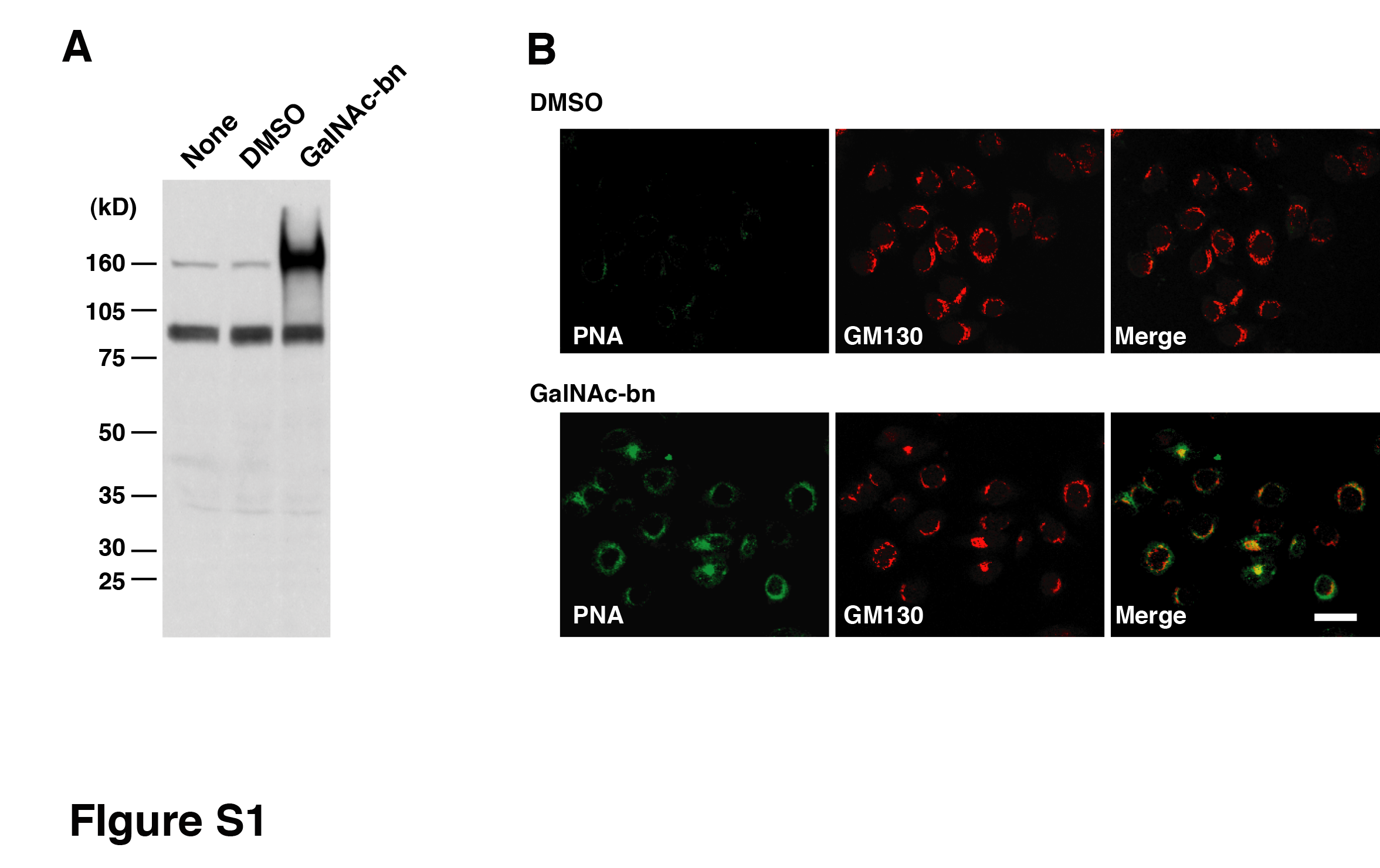

Supplement: Figure S1 — (A) Western blot analysis of the PNA lectin binding levels (a marker for the inhibition of O-glycosylation levels) in Colo 205 cells. Cells were treated with GalNAc-bn for 1 d. (B) Immunoreactivity of PNA lectin overlapped with the localization of the Golgi apparatus in the presence of GalNAc-bn treatment. GalNAc-bn-treated cells were observed 24 h after stimulation. Scale bar: 30 µm. (TIF) [file pone.0069732.s001.tif]

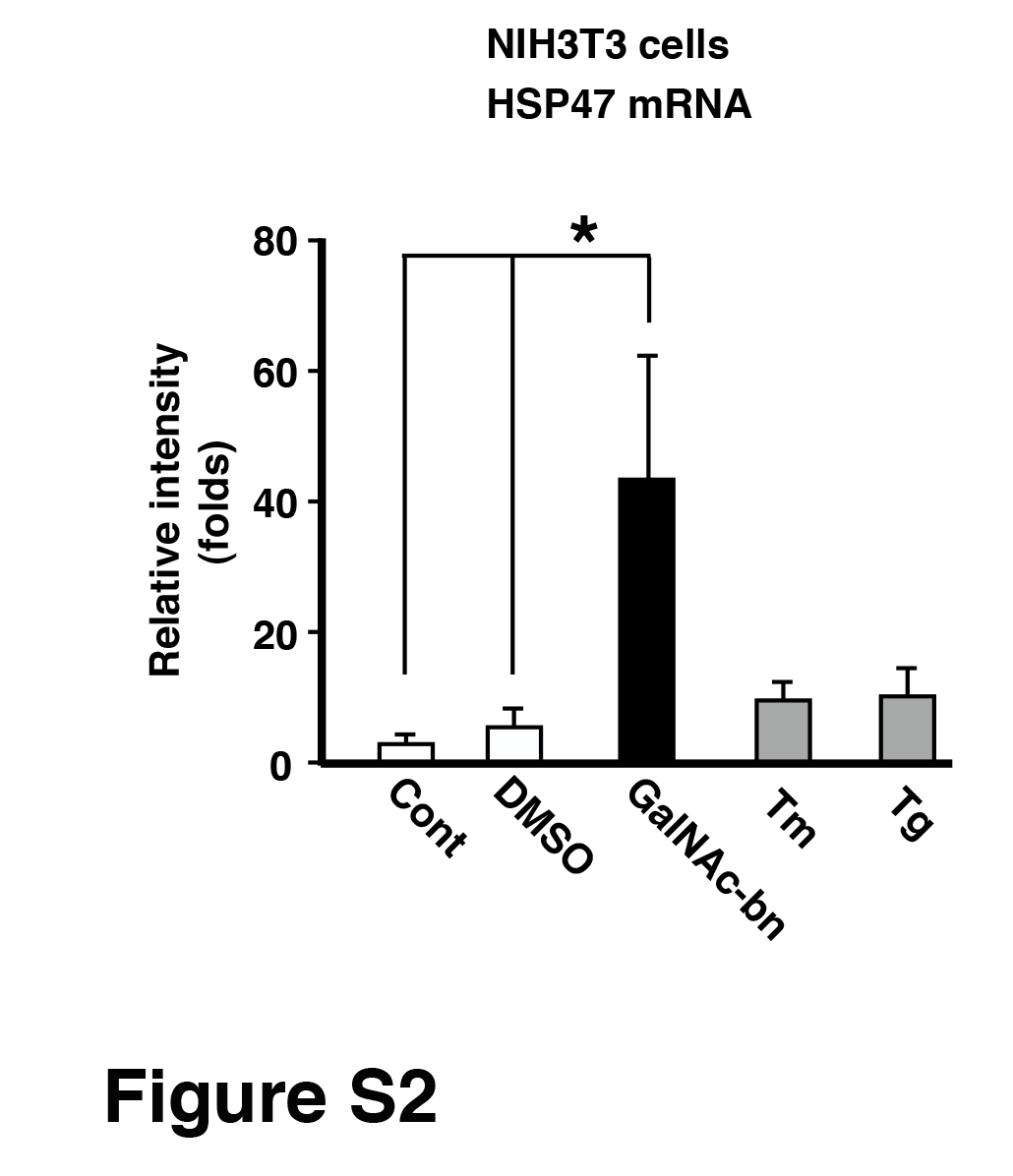

Supplement: Figure S2 — (A, B) Real-time PCR analysis showed increasing HSP47 mRNA levels in Colo 205 cells (A) and NIH3T3 cells (B) specifically after GalNAc-bn, and HSP47 mRNA expressions did not change after Tm, or Tg stimulation. Tm, tunicamycin; Tg, thapsigargin. Data are expressed as the mean ± SEM of at least 3 independent experiments. *p <0.05 (Student’s t test). (TIF) [file pone.0069732.s002.tif]

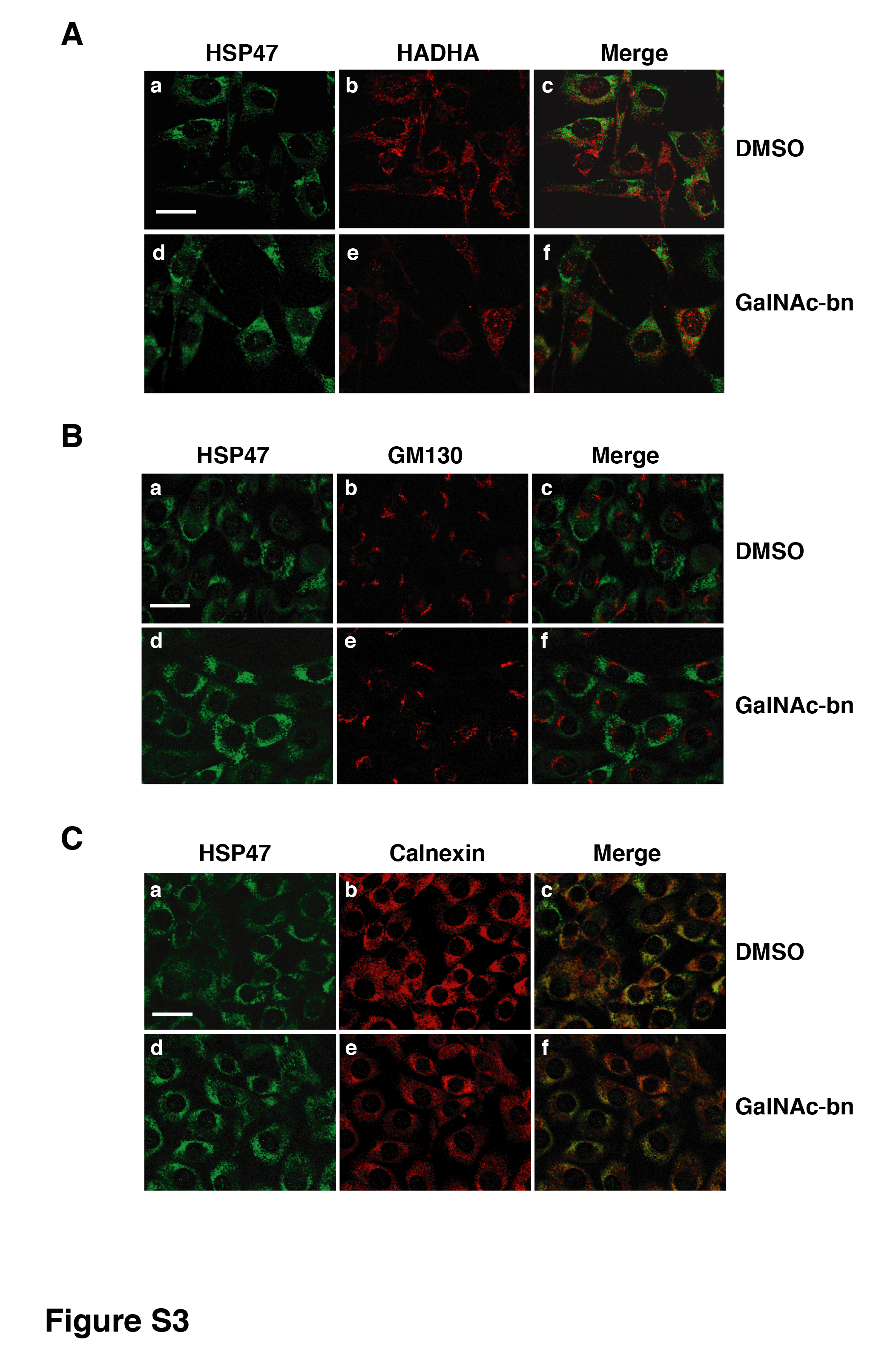

Supplement: Figure S3 — NIH3T3 cells were stained with anti-HSP47 antibodies and anti-HADHA antibodies (mitochondria) (A), anti-GM130 antibodies (Golgi apparatus) (B), and anti-calnexin antibodies (ER) (C) with (d–f) or without (a–c) GalNAc-bn stimulation. GalNAc-bn-treated cells were observed 24 h after stimulation. Scale bar: 20 µm. (TIF) [file pone.0069732.s003.tif]

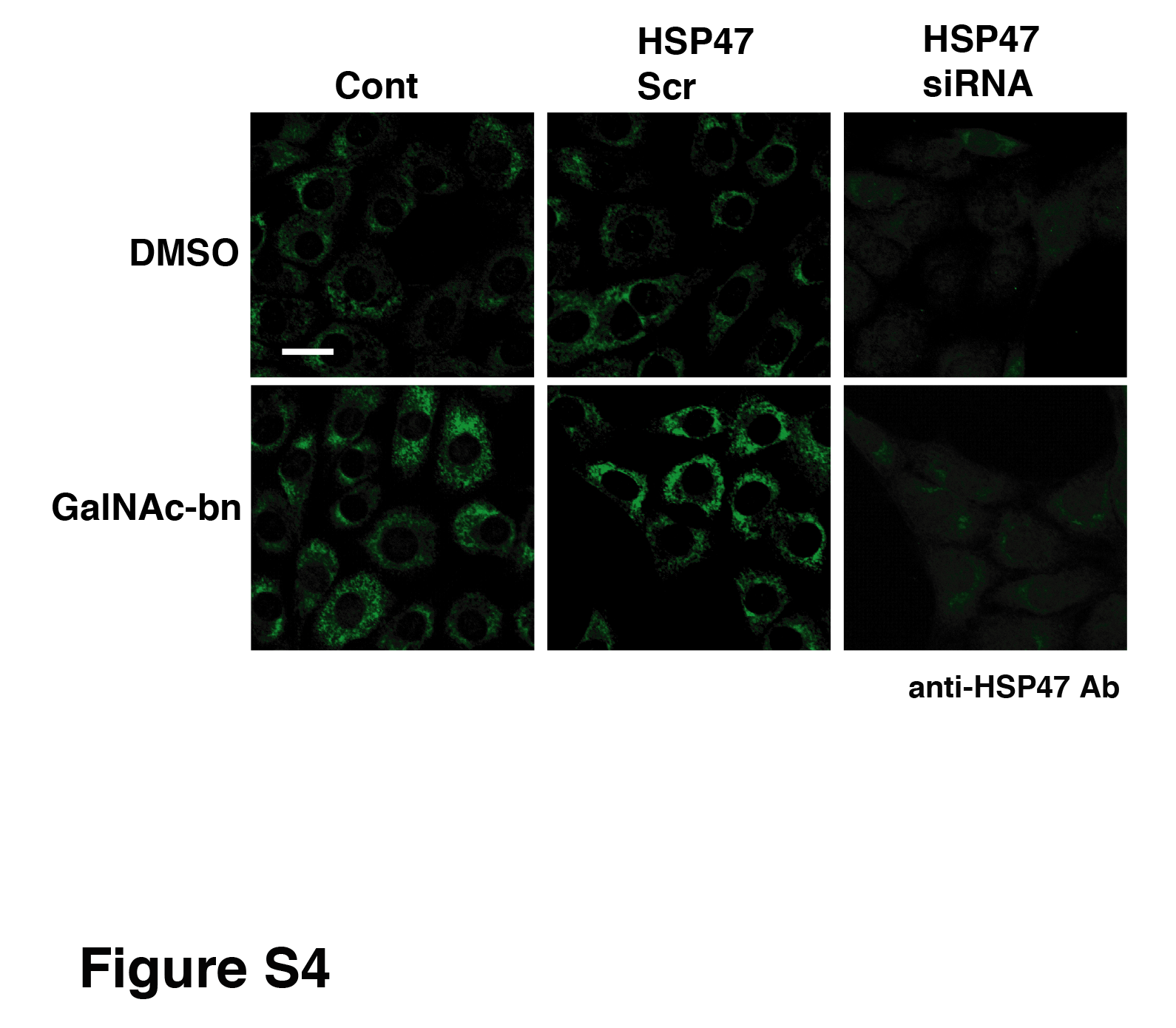

Supplement: Figure S4 — NIH3T3 cells were stained with anti-HSP47 antibodies with (d–f) or without (a–c) GalNAc stimulation. GalNAc-treated cells were observed 24 h after stimulation. Cont, nontransfected cells; Scr, scrambled siRNA-transfected cells; siRNA, HSP47 siRNA-transfected cells. Scale bar: 20 µm. (TIF) [file pone.0069732.s004.tif]

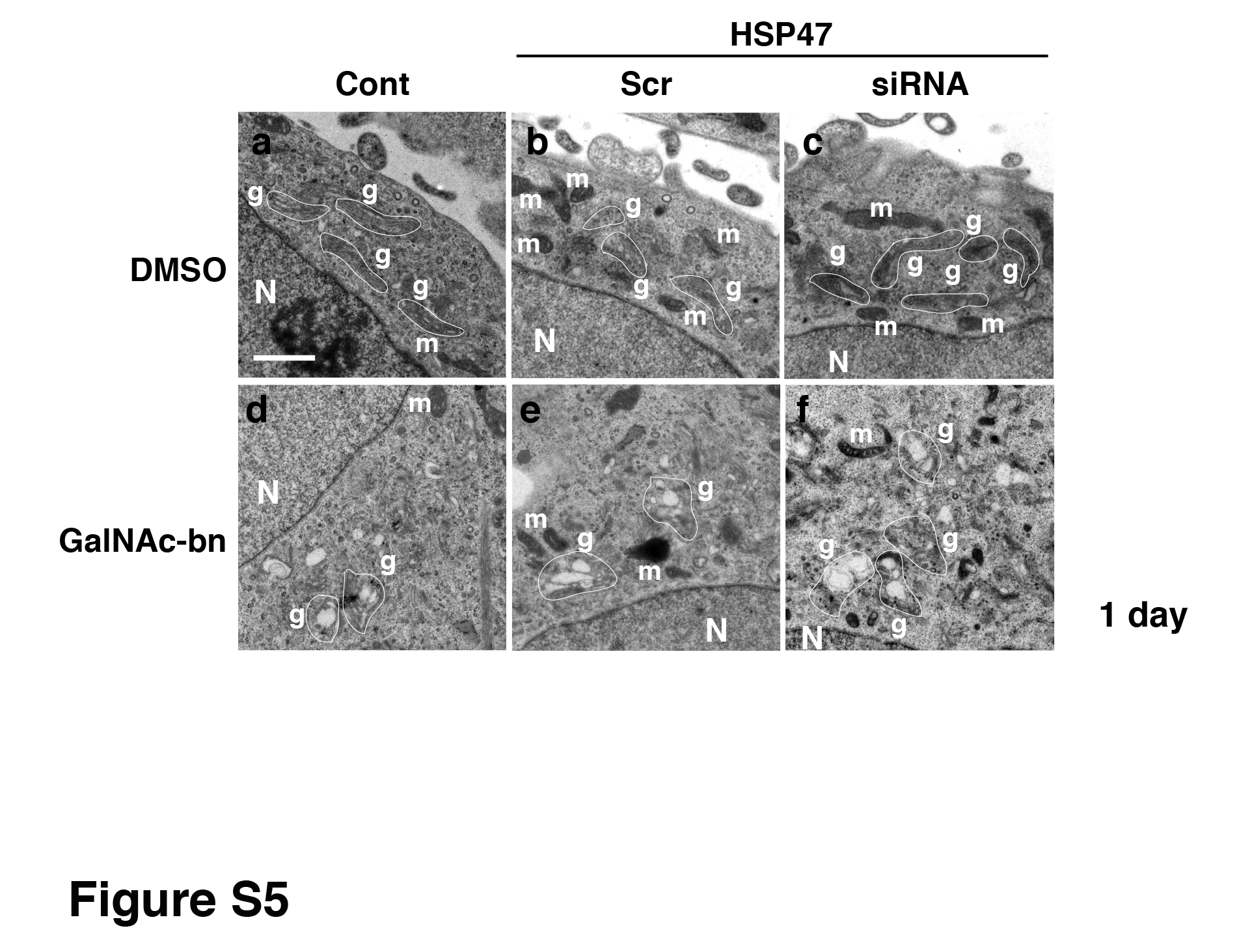

Supplement: Figure S5 — Electron micrographs of NIH3T3 cells 2 d after transfection with scrambled or HSP47 siRNAs and 1 d after treatment with DMSO or GalNAc. GalNAc treatment induced numerous vacuoles around the Golgi apparatus. Cont, untransfected cells; Scr, scrambled siRNA-transfected cells; siRNA, HSP47 siRNA-transfected cells. N, nucleus; g, Golgi apparatus; m, mitochondria; c, primary cilium. Scale bar: 4 µm. (TIF) [file pone.0069732.s005.tif]

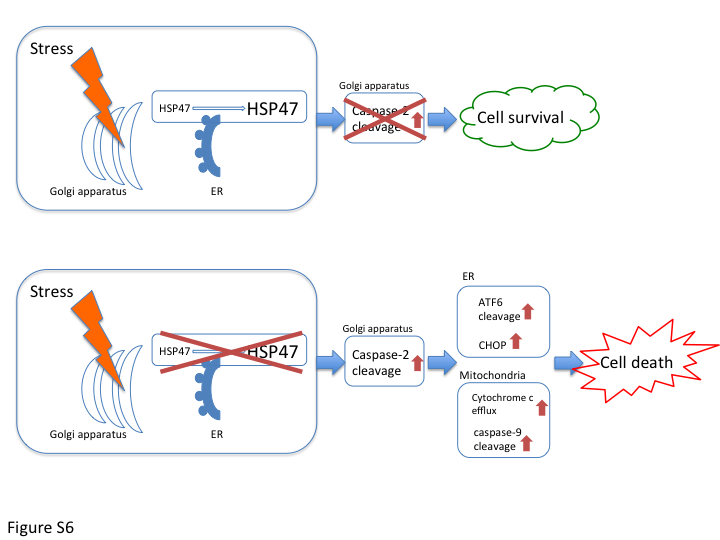

Supplement: Figure S6 — Golgi stress promotes ER-resident chaperone HSP47 expression and protects caspase-2 cleavage. HSP47-knockdown NIH3T3 cells exhibited increased cleavage of Golgi-resident caspase-2. Furthermore, HSP47-knockdown cells exhibited activation of ER-resident unfolded protein response (UPR)-related molecules, and efflux of cytochrome c from the mitochondria to the cytoplasm and activation of mitochondrial caspase-9. Golgi stress influences not only Golgi apparatus function but also ER and mitochondria functions and induced cell death via inhibition of the HSP47. (TIF) [file pone.0069732.s006.tif]
