# Supplementary material for: The Endoplasmic Reticulum-Resident Chaperone Heat Shock Protein 47 Protects the Golgi Apparatus from the Effects of O-Glycosylation Inhibition
Source: PLoS One. 2013 Jul 29;8(7):e69732. doi: 10.1371/journal.pone.0069732 (PMC3726774; doi:10.1371/journal.pone.0069732)
Supplement: File S1 — Extended materials and methods. (DOCX) [file pone.0069732.s007.docx]

**SUPPORTING INFORMATION**

**Extended materials and methods**

**Two-dimensional gel electrophoresis**

Two-dimensional polyacrylamide gel electrophoresis (2DE) was performed using the ZOOM IPG Standard Set (Life Technologies Inc.) according to the manufacturer’s instructions. Cells were lysed in the lysis buffer (1.1x ZOOM 2D Protein Solubilizer 1, 3 mM Tris Base, 1x Protease Inhibitor Cocktail, 20 mM DTT; Life Technologies Corp.). N,N-Dimethylacrylamide (Sigma Chemical Co.) was added to the lysate for alkylation. The lysates were incubated on a rotary shaker for 30 min at room temperature and were solubilized in 165 μL sample rehydration buffer (7 M urea, 2 M thiourea, 2% Chaps, 0.5% ZOOM Carrier Ampholytes, pH 3–10 (Life Technologies), 20 mM DTT, and 0.1% bromophenol blue). Prior to isoelectric focusing (IEF), ZOOM strips (pH 3–10) NL (Life Technologies Corp.) were incubated in a rehydration solution containing the sample for 16 h, and IEF was conducted using the step voltage of 200 V (20 min), 450 V (15 min), 750 V (15 min), and 2000 V (30 min). After IEF, the strips used for gel electrophoresis were first equilibrated in 4.5 mL lithium dodecyl sulfate (LDS) sample buffer along with 0.5 mL of 10× sample reducing agent (Life Technologies Corp.), and subsequently in the same solution containing 125 mM iodoacetamide without reducing agent (15 min each). Samples were separated in the second dimension on NuPAGE Novex 4–12% Bis–Tris ZOOM gels in MOPS SDS running buffer (Life Technologies Corp.), and proteins were stained with 2D-SILVER STAIN II (Daiichi Pure Chemicals, Tokyo, Japan).

**In-gel digestion and mass spectrometry**

After 2DE, spots of various intensities were excised from polyacrylamide gels. These spots were destained and underwent an in-gel 16 h tryptic digestion at 37°C. The resulting peptides were extracted from the gel with a 50% (v/v) acetonitrile (AcN) and 1% (v/v) TFA solution. A 1-μL aliquot was spotted onto the AnchorChip targets (Bruker Daltonik, Bremen, Germany) with 1 μL of matrix (α-cyano-4-hydroxcinnamic acid, 8 mg/mL in 50% (v/v) AcN, 1% (v/v) TFA) and allowed to air dry. Mass spectra of tryptic peptides were obtained using an UltraReflex MALDI-TOF MS (Bruker Daltonik). The obtained peptide mass fingerprints (PMFs) were processed in FlexAnalysis (Bruker Daltonik) and used to identify the corresponding proteins in Biotools ver. 2.2 (Bruker Daltonik), which triggered a Mascot (Matrix Science, MA, USA) search.

**Supporting Information Legends**

**Figure S1. GalNAc-bn treatment decreases O-glycosylation in Colo 205 cells.** (A) Western blot analysis of the PNA lectin binding levels (a marker for the inhibition of O-glycosylation levels) in Colo 205 cells. Cells were treated with GalNAc-bn for 1 d. (B) Immunoreactivity of PNA lectin overlapped with the localization of the Golgi apparatus in the presence of GalNAc-bn treatment. GalNAc-bn-treated cells were observed 24 h after stimulation. Scale bar: 30 μm.

**Figure S2. Golgi stress induced elevation of HSP47 mRNA expression levels.** (A, B) Real-time PCR analysis showed increasing HSP47 mRNA levels in Colo 205 cells (A) and NIH3T3 cells (B) specifically after GalNAc-bn, and HSP47 mRNA expressions did not change after Tm, or Tg stimulation. Tm, tunicamycin; Tg, thapsigargin. Data are expressed as the mean ± SEM of at least 3 independent experiments. *p <0.05 (Student’s t test).

**Figure S3. Intracellular localization of HSP47 in NIH3T3 cells.** NIH3T3 cells were stained with anti-HSP47 antibodies and anti-HADHA antibodies (mitochondria) (A), anti-GM130 antibodies (Golgi apparatus) (B), and anti-calnexin antibodies (ER) (C) with (d–f) or without (a–c) GalNAc-bn stimulation. GalNAc-bn-treated cells were observed 24 h after stimulation. Scale bar: 20 μm.

**Figure S4. HSP47 protein expression check by immunocytochemistry.** NIH3T3 cells were stained with anti-HSP47 antibodies with (d–f) or without (a–c) GalNAc stimulation. GalNAc-treated cells were observed 24 h after stimulation. Cont, nontransfected cells; Scr, scrambled siRNA-transfected cells; siRNA, HSP47 siRNA-transfected cells. Scale bar: 20 μm.

**Figure S5. Golgi stress induces the disassembly of the Golgi apparatus in HSP47 siRNA-transfected NIH3T3 cells.** Electron micrographs of NIH3T3 cells 2 d after transfection with scrambled or HSP47 siRNAs and 1 d after treatment with DMSO or GalNAc. GalNAc treatment induced numerous vacuoles around the Golgi apparatus. Cont, untransfected cells; Scr, scrambled siRNA-transfected cells; siRNA, HSP47 siRNA-transfected cells. N, nucleus; g, Golgi apparatus; m, mitochondria; c, primary cilium. Scale bar: 4 μm.

**Figure S6. Hypothetical pathways by which Golgi stress induces cell death of NIH3T3 cells.** Golgi stress promotes ER-resident chaperone HSP47 expression and protects caspase-2 cleavage. HSP47-knockdown NIH3T3 cells exhibited increased cleavage of Golgi-resident caspase-2. Furthermore, HSP47-knockdown cells exhibited activation of ER-resident unfolded protein response (UPR)-related molecules, and efflux of cytochrome c from the mitochondria to the cytoplasm and activation of mitochondrial caspase-9. Golgi stress influences not only Golgi apparatus function but also ER and mitochondria functions and induced cell death via inhibition of the HSP47.
